# Direct Detection of Hydrogen Bonds in Supramolecular Systems Using ^1^H–^15^N Heteronuclear Multiple Quantum Coherence Spectroscopy

**DOI:** 10.1021/jacs.2c10742

**Published:** 2022-12-12

**Authors:** Michael A. Jinks, Mark Howard, Federica Rizzi, Stephen M. Goldup, Andrew D. Burnett, Andrew J. Wilson

**Affiliations:** † School of Chemistry, University of Leeds, Woodhouse Lane, Leeds LS2 9JT, U.K.; ‡ Department of Chemistry, University of Southampton, Highfield Campus, Southampton SO17 2BJ, U.K.; § Astbury Centre for Structural Molecular Biology, University of Leeds, Woodhouse Lane, Leeds LS2 9JT, U.K.

## Abstract

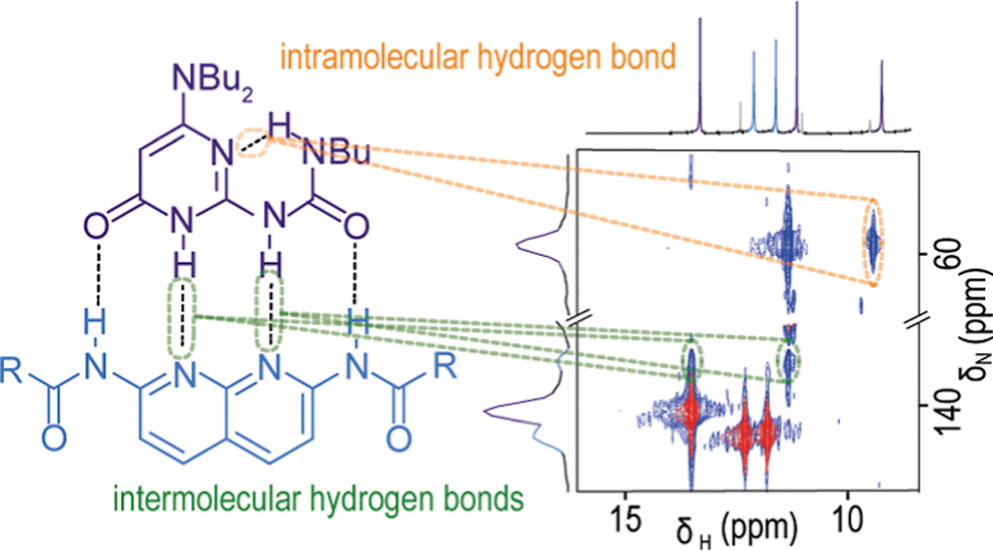

Hydrogen-bonded supramolecular systems are usually characterized in solution through analysis of NMR data such as complexation-induced shifts and nuclear Overhauser effects (nOe). Routine direct detection of hydrogen bonding particularly in multicomponent mixtures, even with the aid of 2D NMR experiments for full assignment, is more challenging. We describe an elementary rapid ^1^H–^15^N HMQC NMR experiment which addresses these challenges without the need for complex pulse sequences. Under readily accessible conditions (243/263 K, 50 mM solutions) and natural ^15^N abundance, unambiguous assignment of ^15^N resonances facilitates direct detection of intra- and intermolecular hydrogen bonds in mechanically interlocked structures and quadruply hydrogen-bonded dimers—of dialkylaminoureidopyrimidinones, ureidopyrimidinones, and diamidonaphthyridines—in single or multicomponent mixtures to establish tautomeric configuration, conformation, and, to resolve self-sorted speciation.

## Introduction

Hydrogen bonding is a “master-key” non-covalent interaction for supramolecular assembly.^1−3^ The interplay of multiple interactions,^4−8^ configuration and conformational preferences,^4,9−13^ alongside secondary electrostatic interactions^14−16^ to tune hydrogen-bonding strength,^17,18^ has furnished design rules for hydrogen-bonding motifs^19−21^ and given rise to several that form strongly associated dimers that are widely employed in materials science.^20,22,23^

Hydrogen bonding can be inferred in the solid state using X-ray crystallography or neutron diffraction.^24−27^ Solution-based techniques (UV/vis, NMR, and IR) can qualitatively establish that molecular recognition takes place by changes in resonance or vibrational frequency, (e.g., through complexation-induced shifts in 1D NMR) or intercomponent proximity (e.g., through nOe), while titration or dilution experiments can provide thermodynamic parameters.^28^ However, while a change in the resonant frequency may indicate hydrogen bonding, this represents a consequence, rather than direct observation of, the hydrogen bond. Methods for direct detection of hydrogen bonds in solution are advantageous as they allow rapid assignment of structures (rather than proximity) in bound complexes, to complement solid-state studies. For analyses of biomolecule folding and assembly by NMR, several techniques (IMPACT-HMNBC, *J*_NN_HNN-COSY, SOFAST-HMBC, SOFAST-HMQC, etc.)^29−36^ have been employed, typically using ^15^N isotope-enriched samples. Some 2D NMR methods have also been shown to be amenable to structural studies at ^15^N natural abundance.^37^ Although synthetic ^15^N isotope-enriched supramolecular synthons have been studied using these techniques^38^ and solid-state methods,^39^ such analyses are scarce. In contrast to the ready access to ^15^N labeled proteins afforded through expression in ^15^N enriched media, it is more challenging and costly, to incorporate ^15^N through chemical synthesis. Despite clear advantages, the low abundance of ^15^N (isotopic abundance = (3.46–4.21) × 10^–3^ molar fraction) renders development of spectroscopic methods that do not rely on enrichment, desirable for identification of hydrogen bonds. This need is further emphasized by the considerable effort currently devoted to the study of multicomponent self-sorting assemblies^40−46^ and system chemistry,^47−49^ where characterization of speciation is challenging. In this work, we demonstrate that *J* coupling between hydrogen-bonded nitrogen and hydrogen nuclei in heteronuclear multiple quantum coherence (HMQC) experiments allows observation of intra and intermolecular hydrogen bonds in diverse supramolecular architectures, for example, rotaxanes and hydrogen-bonded homo-/heterodimers. The method provides insights on the co-conformation in rotaxanes and tautomeric configuration, conformation, and speciation in self-sorted mixtures of hydrogen-bonded motifs.

## Results and Discussion

**Figure 1:**
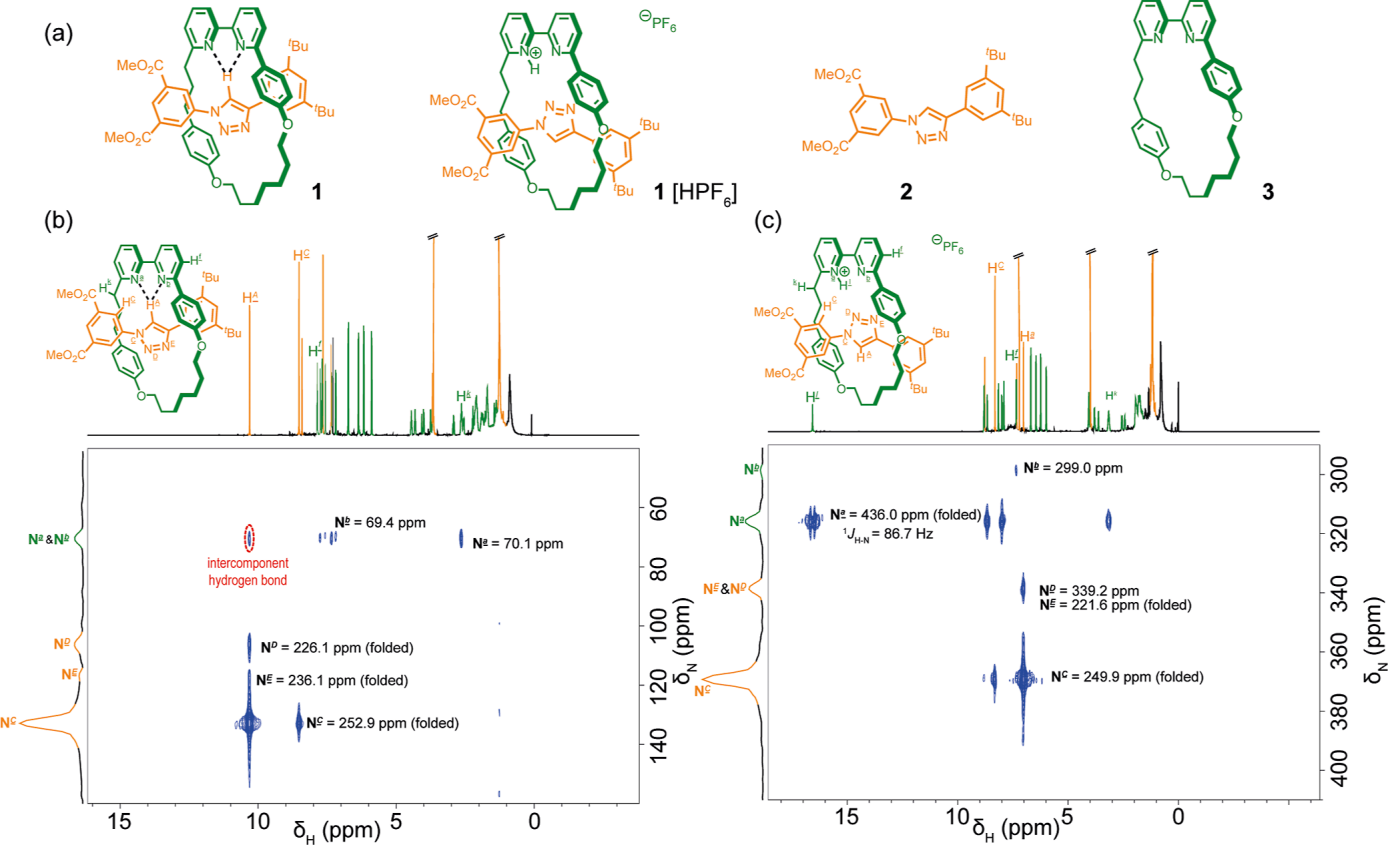
^1^H–^15^N HMQC spectra (500 MHz-51 MHz, CDCl_3,_ 263 K, 50 mM) of mechanically interlocked architectures; (a) structures of [2]rotaxane **1**, [2]-rotaxane **1**[HPF_6_] axle **2** and macrocycle **3**, (b) [2]-rotaxane **1**; (c) protonated [2]-rotaxane **1**[HPF_6_]; dotted black lines indicate intercomponent hydrogen bonds. Highlighted spin systems denote those identified by *nJ* cross couplings. The ^1^H projection is the 1D NMR spectra.

We first used a rapid ^1^H–^15^N HMQC experiment to observe hydrogen bonding in [2]-rotaxane **1** (Figure 1a), which contains two unique bipyridine nitrogen environments,^50^ prepared via active template CuAAC chemistry (see Supporting Information, Figure S1).^51−53^ Rotaxanes are mechanically interlocked structures, in which an axle is threaded through a macrocycle. Dissociation is blocked by sterically large “stoppering” units.^54^ The steric crowding imposed by the mechanically interlocked thread and ring enforces non-covalent interactions between the two regardless of solvent polarity, making interlocked structures useful platforms for probing through space interactions.^55^ The elementary approach utilized an HMQC pulse sequence from the Bruker preset sequence (Figure S2) with 1/2*J* delay set to 0.0625 s (*J* = 8 Hz) to utilize *nJ* couplings in the polarization transfer between nuclei. The combined relaxation delay and acquisition time was set at 650 ms to obtain a fast repetition rate without detrimental loss of signal to noise. SOFAST sequences with repetition rates of 0.3–0.4 s did not detect long-range couplings over +10 h of acquisition in our systems. Measurements were typically acquired using a standard TBO or a TXI probe operating at 263 K to minimize molecular motion between the axle and macrocycle components. The elementary nature of this approach derives from the optimization of an experiment on many Bruker spectrometers. This approach can easily be transferred to spectrometers from other manufacturers.

In addition to the ^1^H–^15^N HMQC spectrum for rotaxane **1** (Figure 1a,b), spectra for the axle **2** and macrocycle **3** components were obtained to account for through bond correlations of non-hydrogen-bonded spin systems (see Supporting Information Figure S3–S4). To maximize F1 resolution, the ^15^N chemical shift window was restricted between 40 and 160 ppm, *nJ* NH long-range correlations that were deshielded outside this window are aliased in F1 as a consequence of the echo-antiecho selection used in the gradient HMQC sequence. Such spectral folding is observed in all spectra (see Supporting Information for further details and Figure S5 as an example). A downfield shift for the triazole proton H^*A*^ from 8.24 to 10.30 ppm was observed for the rotaxane relative to the component axle **2** indicative of hydrogen bonding (see Supporting Information, Figure S1). All five nitrogen environments for rotaxane **1** were observed (Figure 1b) and could be assigned using 1 and 2D NMR (Table 1). Correlation between proton H^*A*^ on the triazole and bipyridine nitrogen atoms N^*a*^ and N^*b*^ is observed, indicating through-space interaction. This through-space correlation signifies direct orbital communication between the hydrogen and nitrogen atoms, that is, a proximity-enforced hydrogen bond. The two nitrogen atoms in the bipyridine are inequivalent and move upfield relative to macrocycle **3**. Prior solid-state studies on mechanically interlocked architectures incorporating macrocycle **3** imply preferential hydrogen-bonding of H^*A*^ to N^*a*^;^56−62^ however, a preference cannot be established on the basis of this NMR experiment; the correlation between H^*A*^ and N^a^/N^*b*^ could be observed at higher temperatures although in general, loss of signal occurred at higher temperatures (see Supporting Information Figure S6–S8).

**Table 1:** Chemical Shift Values for Nitrogen Atoms in Compounds **1**, **1**[HPF_6_], **2**, and **3** (in ppm)

	N^*a*^	N^*b*^	N^*C*^	N^*D*^	N^*E*^
**1**	70.1	69.6	252.9	226.1	236.1
**1**[HPF_6_]	436.0	299.0	249.9	339.2	221.6
**2**			371.8	230.0	240.0
**3**	189.2	183.1			

Protonation of rotaxane **1** to generate [2]-rotaxane **1**[HPF_6_] induces a co-conformational change, providing a further opportunity to assess the use of the ^1^H–^15^N HMQC experiment to identify stimuli-induced structural changes. Upon protonation of [2]-rotaxane **1** to generate **1**[HPF_6_], the resonance for H^*A*^ moves upfield to 7.02 ppm as it no longer participates in hydrogen bonding (Figure 1c). A new resonance appears at 16.59 ppm corresponding to the bipyridinium proton H^*l*^ (Figure S2d). In the ^1^H–^15^N HMQC spectra for **1**[HPF_6_], all five nitrogen environments are observed (Table 1, see also Figure S7). Bipyridine nitrogen atoms N^*a*^ and N^*b*^ move downfield relative to the unprotonated macrocycle, in particular N^*a*^, indicating protonation. The cross peak for H^*l*^ resolves as a doublet (see also Figure S4ii), with a 1*J* coupling constant of 86.7 Hz, typical of 1*J* values observed in enriched systems, possibly as a consequence of a short NH bond.^32,63,64^ The experiment thus unambiguously establishes the site of protonation, which is in agreement with that proposed for a previously reported solid-state structure of a related rotaxane.^57^ No correlation was observed between H^*l*^ and the nitrogen atoms of the triazole. Hydrogen bonding was implied between the bipyridinium macrocycle and neutral axle in previously reported X-ray structures.^57,65^ The hydrogen bond may not be observed here due to chemical exchange line broadening on either the *J*-coupling, transverse relaxation rate (R2), or chemical shift difference time scales. Alternatively, the dominant structure in solution may differ from that observed in the solid state (e.g., cation−π interactions between pyridinium and triazole may compete with the expected H-bond), or rapid exchange between different hydrogen-bonded states may take place. Repeating the experiment at lower temperature (243 K) and exploring a range of different 1/2*J* settings failed to identify a correlation associated with hydrogen bonding. However, non-covalent interactions between the bipyridinium proton and N^*b*^ and/or N^*D*^ seem likely, given that δN^*b*^ and δN^*D*^ for **1**[HPF_6_] exhibit significant downfield chemical shift differences relative to unprotonated rotaxane **1** (Δδ of −229.4 and −113.1 ppm, respectively).

The ^1^H–^15^N HMQC experiment was then applied to quadruply hydrogen-bonded complexes^4,66−70^ of dialkylaminoureidopyrimidinones (AUPy) **4**, ureidopyrimidinones (UPy) **5**, and diamidonaphthyridines (DAN) **6** bearing solubilizing modifications on AUPy **4** and UPy **5** (see the Supporting Information). AUPy, UPy, and DAN motifs form strongly hydrogen-bonded dimers (*K*_a_ > 10^5^ M^–1^ in chloroform)^4,62−66^ and exhibit well-defined self-sorting behavior in three component systems,^66^ making them ideal models to test the capability of the rapid ^1^H–^15^HMQC experiment. Because the hydrogen-bonded dimers contain hydrogen atoms directly bound to nitrogen atoms, two experiments were performed for each sample—one where acquisition settings resulted only in observation of the 1*J* couplings and another where 1*J* and *nJ* (i.e., single bond and long-range correlations) were observed which allowed the later to be readily distinguished and facilitated characterization of hydrogen-bonded dimers.

**Figure 2:**
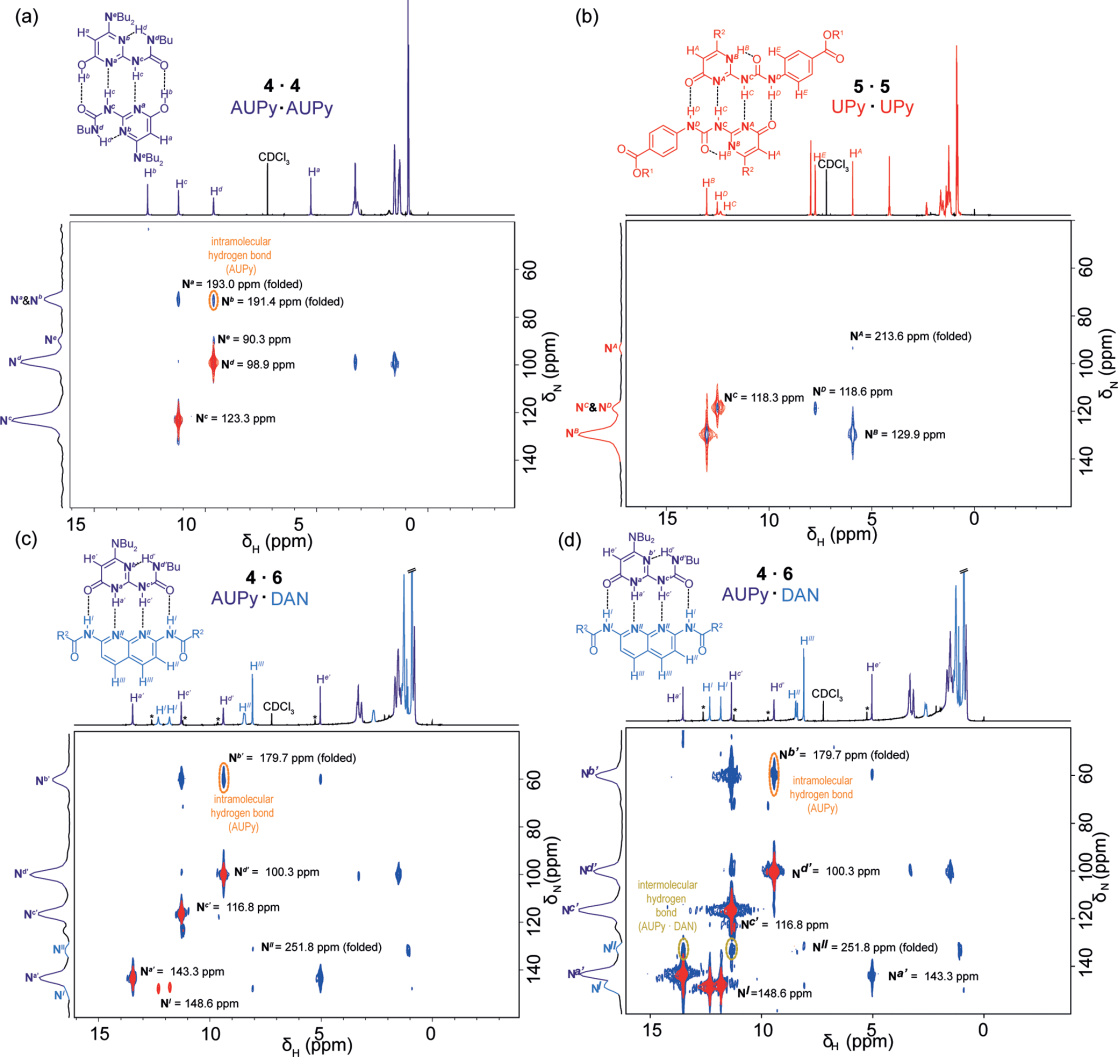
^1^H–^15^N HMQC spectra of hydrogen-bonding motifs (500–51 MHz, CDCl_3_, 50 mM); (a) AUPy·AUPy (**4** · **4** at 263 K); (b) UPy·UPy (**5** · **5** at 263 K); (c) AUPy·DAN (**4** · **6** at 263 K); and (d) AUPy·DAN (**4** · **6** at 243 K), peaks indicated by an asterisk (*) correspond to excess AUPy **4**; red correlations arise from 1*J* couplings, and blue correlations arise from *nJ* couplings. Implicit hydrogen atoms indicate the hydrogen-bonded atoms. The dotted black lines indicate hydrogen bonds. R^1^ = 2-ethlyhexyl and R^2^ = −CH(Et)(Bu). The ^1^H projection is the corresponding 1D NMR spectra.

For the AUPy·AUPy dimer (**4** · **4**) (Figures 2a and S7–S8), 1*J* couplings to N^*c*^ = 123.3 ppm and N^*d*^ = 98.9 ppm and *nJ* couplings to N^*a*^ = 193.0 ppm, N^*b*^ = 191.4 ppm, and exocyclic nitrogen N^*e*^ = 90.3 ppm were observed. Correlation between the hydrogen attached to N^*d*^ and the protons on the alkyl chain of the urea side chain was also observed. The results are consistent with the preferred configuration described previously (i.e., self-associated pyrimidinol tautomer).^66,67^ Enol proton H^*b*^ is directly bound to an oxygen and hydrogen-bonded to a carbonyl oxygen atom thus has no observable correlations. H^*d*^ correlates with N^*b*^; through-space communication between orbitals thus indicates intramolecular hydrogen bonding. Proton H^*c*^ correlates to N^*a*^; while an intermolecular hydrogen bond is implied in X-ray crystal structures,^66^ coupling arising from intermolecular hydrogen bonding and intramolecular coupling to N^*a*^ are degenerate.

In the ^1^H–^15^N HMQC spectra for the UPy·UPy dimer (**5** · **5**) (Figure 2b and S9), four nitrogen atoms were detected, with 1*J* couplings to N^*B*^ = 129.9 ppm, N^*C*^ = 118.3 ppm, and N^*D*^ = 118.6 ppm, with N^*A*^ = 213.6 ppm identified in the *nJ*^1^H–^15^N HMQC spectra. The spectra also highlight challenges presented by peak broadening for H^*C*^ which result in a weaker than expected correlation to N^*C*^. No correlation between N^*A*^ and H^*C*^ was observed under the conditions of this experiment, although such a correlation could be observed at lower temperatures (see later).

^1^H–^15^N HMQC experiments for AUPy·DAN (**4** · **6**) (Figures 2c and S10–S13) identified AUPy nitrogen atoms, N^*a*′^, N^*b*′^, N^*c*′^, and N^*d*′^. The exocyclic nitrogen atom on the di-butyl substituent was not observed, possibly due to signal overlap with N^*b*′^. The most dramatic Δδ_N_ (−49.7 ppm) was observed for N^*a*′^, which switches from a pyrimidinol nitrogen hydrogen-bond acceptor in **4** · **4** to a pyrimidinone hydrogen-bond donor upon interaction with DAN **6**. N^*b*′^ and both urea nitrogen atoms N^*c*′^ (Δδ_N_ = −6.5 ppm) and N^*d*′^ (Δδ_N_ = +1.4 ppm) undergo less significant changes when compared to the δ_N_ observed for those resonances in AUPy·AUPy (**4** · **4**). The intramolecular bond is observed between N^b′^ and H^*d*′^; together with the identification of the pyrimidinone tautomer, this is consistent with the *ADDA* hydrogen bonding array required for strong association with the *DAAD* motif of DAN **6**.^70^ Amide nitrogen atoms N^*I*^ were detected by 1*J* correlation to H^I^, while N^II^ was observed at 251.8 ppm, through *nJ* correlation. Unambiguous detection of an intermolecular hydrogen bond for AUPy·DAN (**4** · **6**) proved elusive at 263 K but was possible at 243 K where signal-to-noise ratios improved and exchange between the two regioisomers slowed on the NMR timescale. The better resolved reduced line widths for resonances in DAN **6** at the lower temperature consequently revealed additional correlations (Figure 2d). Through-space correlations observed between H^*a*′^ and H^*c*′^ to N^II^ can be attributed to an intermolecular hydrogen bond.

**Figure 3:**
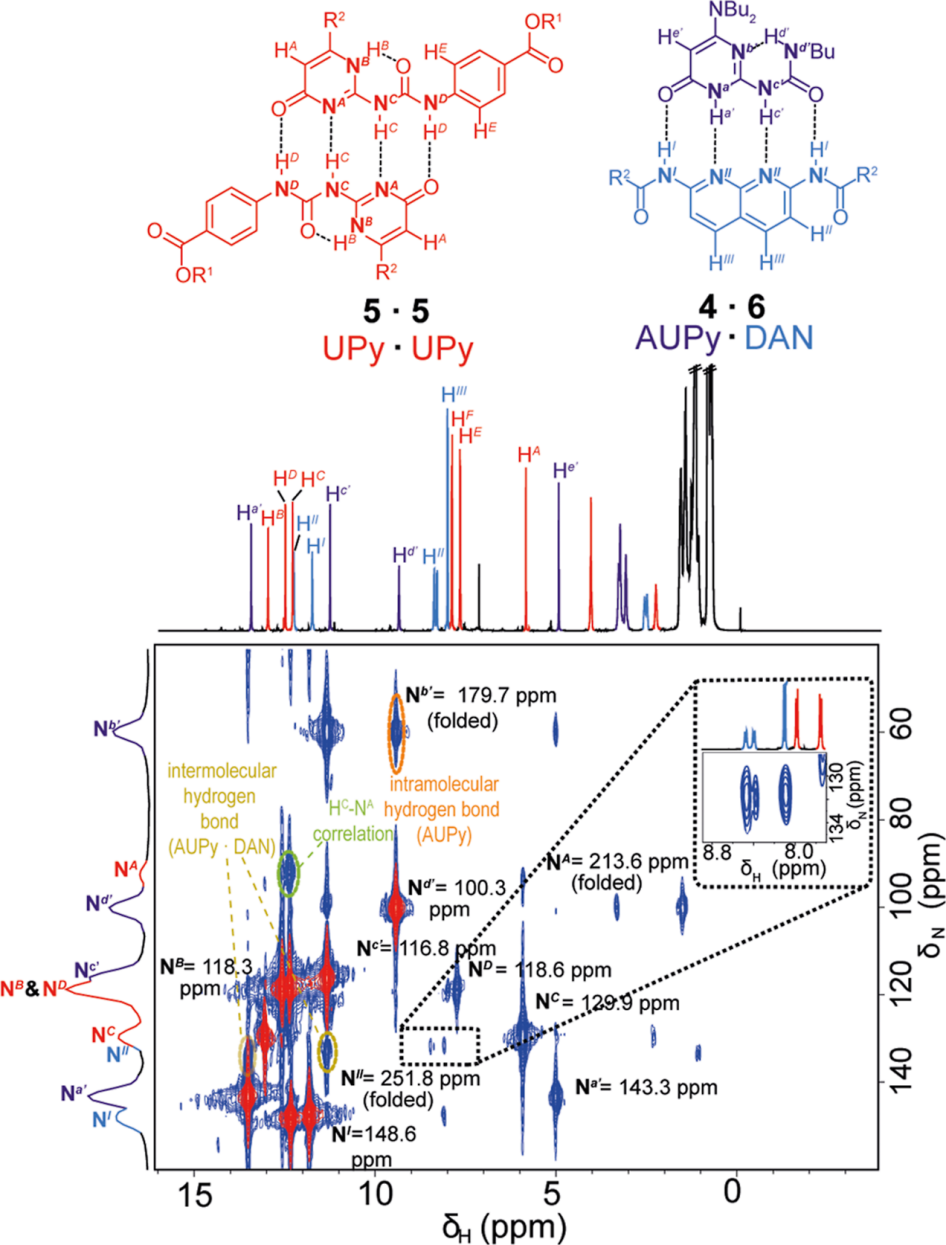
^1^H–^15^N HMQC spectra (500–51 MHz, CDCl_3_, 243 K, 50 mM) of UPy·UPy (**5** · **5**) and AUPy·DAN (**4** · **6**). The red correlations arise form 1*J* couplings, and the blue correlations arise from *nJ* couplings. Emboldened nitrogen atoms indicate the ^15^N atoms detected at natural abundance. The expansion illustrates relevant cross peaks showing desymmetrization of DAN **6**. The dotted lines indicate hydrogen bonds. R^1^ = 2-ethylhexyl and R^2^ = CH(Et)(Bu). The ^1^H projection is the 1D NMR spectra.

A benefit of these experiments is that only hydrogen atoms correlated to nitrogen atoms are observed in the F2 dimension (see the Supporting Information). Therefore for complex mixtures, the ^1^H NMR spectra are simplified to what can be described as “reporter peaks” analogous to PURESHIFT-NMR.^71^ This reduction in the number of resonances simplifies analysis of mixtures, as shown by the ^1^H–^15^N HMQC of a simple self-sorted mixture of 1:1:1 AUPy **4**, DAN **6**, and UPy **5**, which preferentially forms AUPy·DAN (**4** · **6**) and UPy·UPy (**5** · **5**). The experiment was performed at 243 K, to maximize the population of hydrogen-bonded dimers, with the narrowest peak widths, allowing the observation of intra- and intermolecular hydrogen bonds (Figure 3). It should be noted, however, that spectra obtained at 263 K still allow speciation to be determined (see the Supporting Information). All observed cross peaks are in agreement with the AUPy·DAN and UPy·UPy speciation, including a new correlation between N^*A*^ and H^*C*^ for the UPy·UPy dimer as the resonance of H^*C*^ appears as a well-resolved singlet at the lower temperature. Such analyses usually require multiple spectra to assign and identify shifted resonances, whereas the diagnostic cross peaks allow this here in a single experiment.

## Conclusions

In conclusion, we have shown that a rapid ^1^H–^15^N HMQC experiment allows for the first time direct observation of inter- and intramolecular hydrogen bonds to nitrogen in multiple supramolecular architectures including interlocked architectures and hydrogen-bonded dimers, at natural ^15^N abundance and readily accessible temperatures, where the solvent choice optimizes inter- and intramolecular hydrogen bonding. Our results show that the experiment is sensitive to temperature, indicating the exchange dynamics of the hydrogen-bonded proton (i.e., exchange line broadening on either the *J*-coupling, transverse relaxation rate (R2), or chemical shift difference time scales) influence the observed correlations. However, the method should be practical on many spectrometers and can resolve conformational and tautomeric configuration. The experiment is likely to be particularly powerful in deconvoluting complex systems comprising multiple different hydrogen-bonded motifs to resolve speciation.^46,66^ Taken together, our data demonstrate the broad utility of this rapid ^1^H–^15^N HMQC experiment for potential analyses of an extensive array of supramolecular assemblies involving hydrogen bonds to nitrogen, as long as there is a sufficiently high association constant in an appropriate solvent. Future studies will be directed toward harnessing the experiment for more quantitative analyses of such hydrogen-bonded systems and developing approaches to other classes of hydrogen bonds, for example, N–H···O=C.^72^

## Supporting Information Available

Additional 2D NMR and experimental synthesis and characterization of all compounds (PDF)
