# Supplementary material for: Direct Detection of Hydrogen Bonds in Supramolecular Systems Using 1H–15N Heteronuclear Multiple Quantum Coherence Spectroscopy
Source: J Am Chem Soc. 2022 Dec 12;144(50):23127–33. doi: 10.1021/jacs.2c10742 (PMC9782782; doi:10.1021/jacs.2c10742)
Supplement: Supplementary file 1 — ja2c10742_si_001.pdf [file ja2c10742_si_001.pdf]

Direct Detection of Hydrogen Bonds in Supramolecular Systems Using ^{1}H-^{15}N *H*eteronuclear *M*ultiple *Q*uantum *C*oherence Spectroscopy

Electronic Supporting Information

Michael A. Jinks,[a] Mark Howard,[a] Federica Rizzi,[b] Stephen M. Goldup,[d] Andrew D. Burnett[a] and Andrew J. Wilson*[a,c]

[a] School of Chemistry, University of Leeds, Woodhouse Lane, Leeds LS2 9JT, UK

[d] Department of Chemistry, University of Southampton, Highfield Campus, Southampton, SO17 2BJ, UK

[d] Astbury Centre for Structural Molecular Biology, University of Leeds, Woodhouse Lane, Leeds LS2 9JT, UK

Corresponding Author Email

Andrew J. Wilson - School of Chemistry, University of Leeds, Woodhouse Lane, Leeds LS2 9JT, United Kingdom; orcid.org/0000-0001-9852-6366; Email: a.j.wilson@leeds.ac.uk

Contents

Direct Detection of Hydrogen Bonds in Supramolecular Systems Using ^{1}H-^{15}N HMQC 1

NMR Experiments 3

Synthetic Procedures 17

Novel Compound Syntheses 18

1 rac-R_{mp}/S_{mp}-[2]-rotaxane 18

3 Dimethyl 5-(4-(3,5-di-*tert*-butylphenyl)-1*H*-1,2,3-triazol-1-yl)isophthalate 21

S1 2-Ethylhexyl 4-nitrobenzoate 23

S2 2-Ethylhexyl 4-aminobenzoate 25

5 2-Ethylhexyl 4-(3-(6-(heptan-3-yl)-4-oxo-1,4-dihydropyrimidin-2-yl)ureido)benzoate assumed tautomer 27

Literature Compound Syntheses 30

S3 Dimethyl 5-azidoisophthalate 30

S4 ((3,5-Di-*tert*-butylphenyl)ethynyl)trimethylsilane 31

S5 1,3-Di-*tert*-butyl-5-ethynylbenzene 32

S6 2-Amino-6-(dibutylamino)pyrimidin-4-ol 33

4 *N*-[4-(dibutylamino)-6- (*tert*-butyl[diphenyl]silyl)oxypyrimidin-2-yl]-*N'*-butylurea 34

S7 85:15 Ethyl 4-ethyl-3-oxooctanoate-Ethyl (2*Z*)-4-ethyl-3-hydroxyoct-2-enoate (assumed enol geometry) 35

S8 2-Amino-6-(heptan-3-yl)pyrimidin-4(1*H*)-one 36

S9 7-Amino-1,8-Naphthyridin-2(1*H*)-one (Assumed tautomer) 37

S10 *N*-(7,8-Dihydro-7-oxo-1,8-naphthyridin-2-yl)-2-ethyl-hexanamide 38

S11 *N*-(7-chloro-1,8-naphthyridin-2-yl)-2-ethylhexanamide 39

6 *N,N'*-(1,8-naphthyridine-2,7-diyl)bis(2-ethylhexanamide) (1:1 mix of *meso* and chiral diastereoisomers) 40

References 41

NMR Experiments

Spectrometer: 4-channel Bruker AV4-NEO NMR spectrometer operating at 11.75 Tesla (500 MHz ^{1}H) and utilising a 5 mm TXI probe (unless specifically stated otherwise) with z-field gradients. Low temperature operation was facilitated using an external BCU II chiller.

Experiment: Chloroform-d was placed on $CaCl_2$ before being distilled over Linde 5A molecular sieves before use in ^{1}H NMR experiments. Long range couplings between ^{1}H and natural abundance ^{15}N were observed at 263 K using a Bruker standard gradient selected HMQC pulse sequence with parameter and experimental acquisition modifications to operate through heteronuclear zero and double quantum coherence *via* a 1/2*J* delay set to 625 ms (**Figure S2**). The combined relaxation delay and acquisition time was fixed to 650 ms to obtain a fast repetition rate without detrimental loss of signal to noise. Incidentally, SOFAST sequences with repetition rates of 0.3-0.4 s did not detect long-range couplings over +10 hours of acquisition in our systems. The choice of 1/2*J* (*J* = 8 Hz) was considered optimal for observing correlations propagated by small long-range 1-3 Hz $^{n}J_{NH}$ couplings and 80-100 Hz $^{1}J_{NH}$ couplings, whilst minimizing signal losses due to relaxation during polarisation transfer. The observation of $^{1}J_{NH}$ derived resonances enabled additional confirmation of experiment operation. The HMQC experiment was chosen over LR-HSQC options due its efficient optimisation without the need to compensate for artefacts from the evolution of ^{1}H couplings. Furthermore, as the molecules of interest contained limited nitrogen atoms and fewer N-H moieties; the HMQC was acquired over 10.75 hours with 24 F1 points that were linear predicted to 64 points. Low temperature (263 K) operation was utilised with a Bruker BCU II chiller or LN2 evaporator. Both chilling methods were equally viable, and the spectrometer was monitored as stable for 60+ hours using the 'Record' monitoring option within the TopSpin temperature control suite (edte). Always ensure your spectrometer is capable and set-up to operate for extended low temperature periods including the using probe flush-gas and ceramic low-temperature spinners.

To maximise F1 resolution with limited data points, the ^{15}N chemical shift window was restricted between 40-160 ppm, where ^{15}N is externally referenced to ammonia. However, $^{n}J_{NH}$ long-range correlations of interest were expected to be significantly deshielded outside this window with resonance observations expected within the pyridine and polyheteroaromatic region (~250-400 ppm). This is illustrated by 1,8-naphthyridine that provides a single ^{15}N reference of 314.7 ppm. Therefore, in our HMQC experiment, such deshielded resonances are aliased in F1 as the gradient HMQC sequence utilises echo-antiecho selection. The apparent (υ_a) and true (υ_o) resonant frequencies of an aliased peak are given by $\upsilon_a = \upsilon_o - mSW$, where *m* is the integer equal to the number of times the signal has been aliased through a window of spectral width *SW*. Aliasing can be further confirmed by moving the F1 carrier frequency (from 120 ppm to 140-160 ppm) and monitoring the movement of aliased peaks that still comply with the equation given. To prove the interlocked nature of the rotaxane, comparison of the 1D NMR of the individual components shows alteration in peak resonances upon mechanical bond formation.

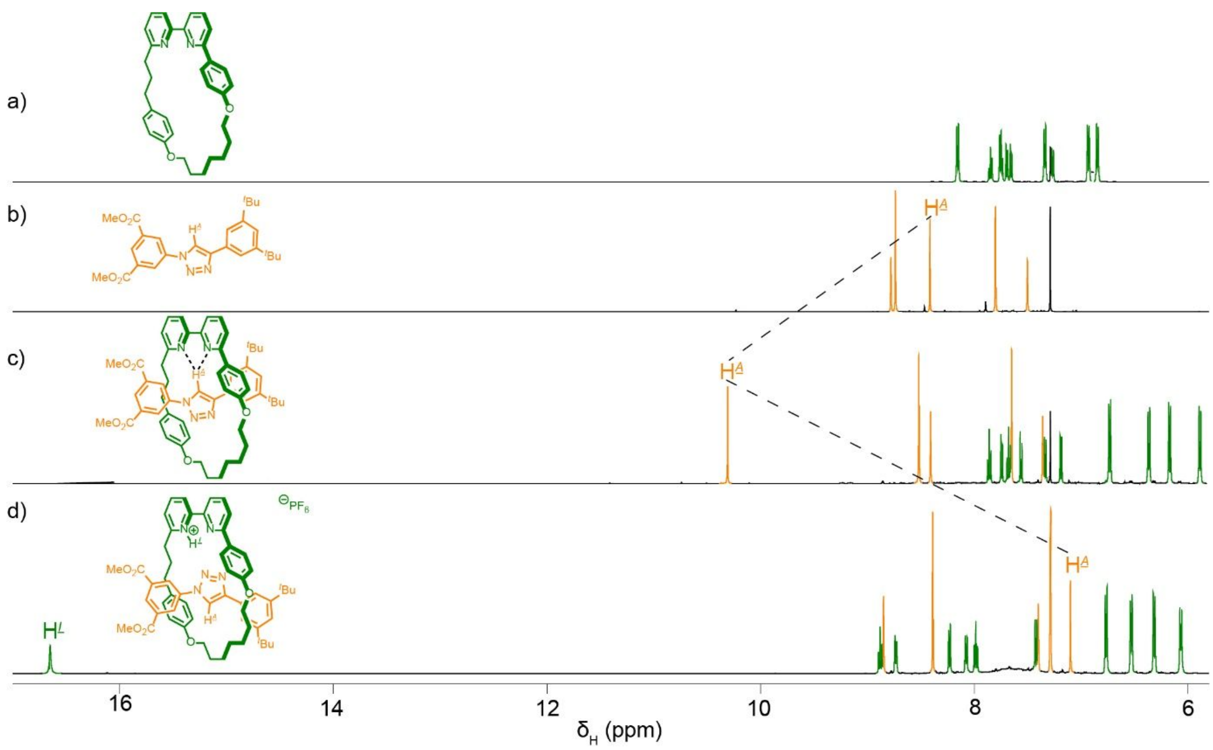

Figure S1. Partial ^{1}H NMR stack plot (500 MHz, $CDCl_3$, 263 K, 50 mM) for a) macrocycle **3**; b) axle **2**; c) rotaxane **1** and: d) rotaxane **1**[HPF_6]. The dotted lines indicate the change in resonance for triazole proton $H^{\underline{A}}$ upon mechanical bond formation and then protonation of the rotaxane.

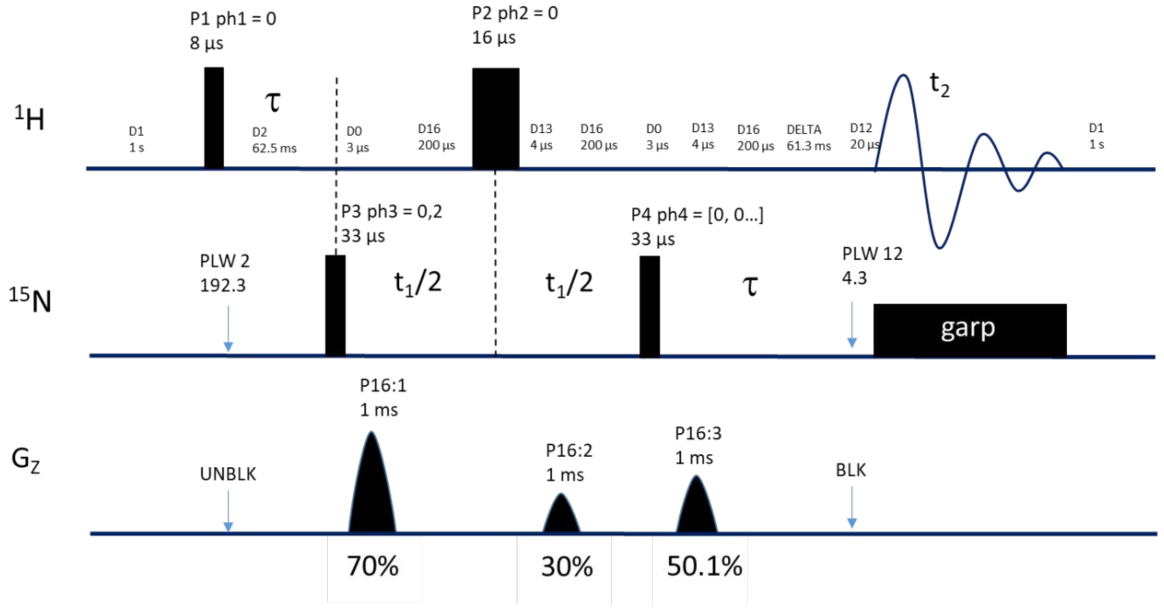

Figure S2. Schematic of the Pulse Sequence for the ^{1}H-^{15}N HMQC NMR experiments via parameter modifications of Bruker pulse sequence **hmqcgpqf.** Pulse lengths and power levels are specific to our Bruker NEO 500 MHz NMR Spectrometer with 5mm TBO z-gradient probe.

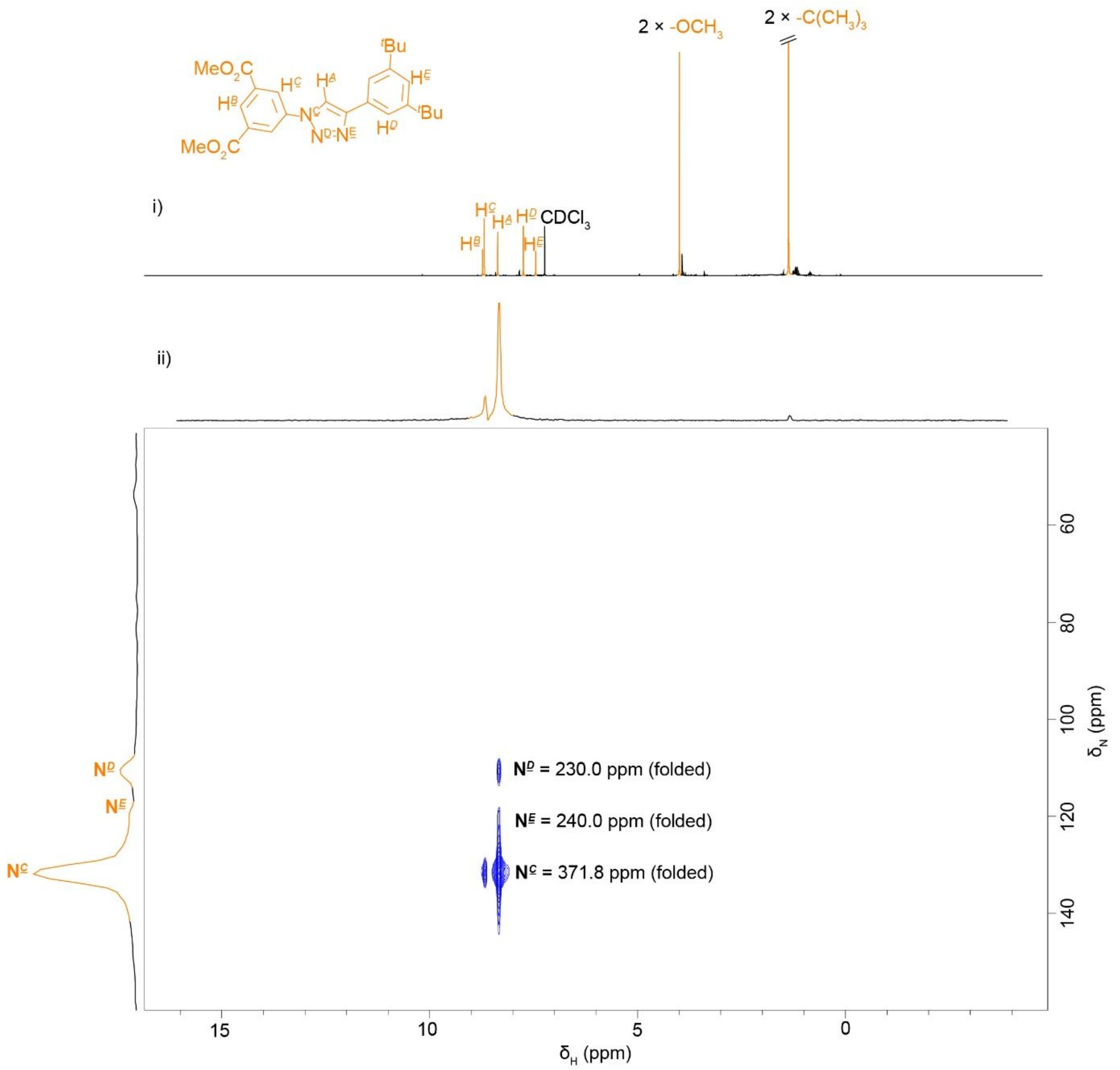

Figure S3. ^{1}H-^{15}N HMQC spectra (500 MHz-51 MHz, $CDCl_3$, 263 K, 50 mM) of axle (**2**). i) The ^{1}H NMR spectra (500 MHz, $CDCl_3$, 263 K, 50 mM) and ii) The F2 projection from the ^{1}H-^{15}N HMQC experiment (500 MHz-51 MHz, $CDCl_3$, 263 K, 50 mM).

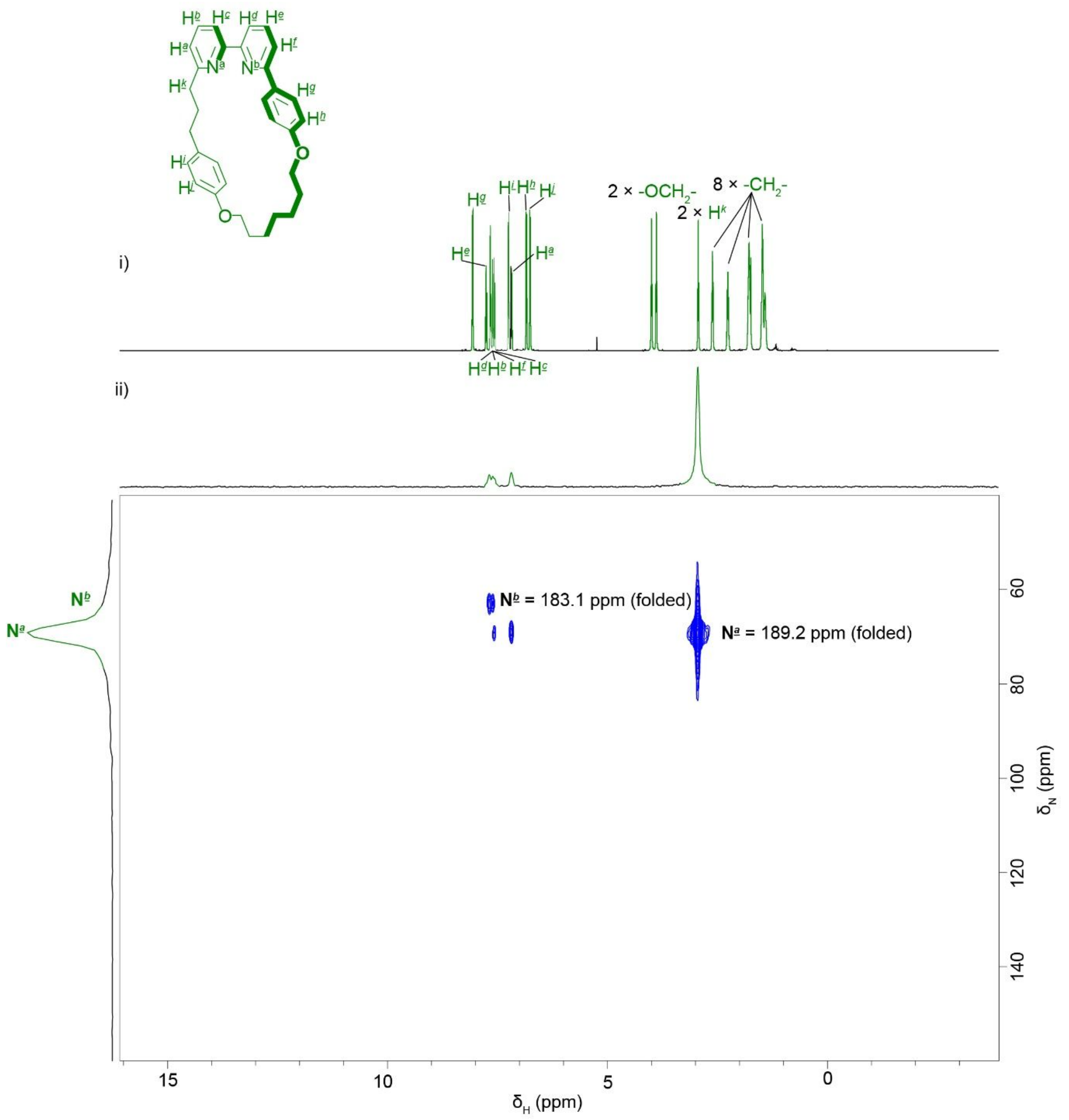

Figure S4. ^{1}H-^{15}N HMQC spectra (500 MHz-51 MHz, $CDCl_3$, 263 K, 50 mM) of macrocycle (**3**). i) The ^{1}H NMR spectra (500 MHz, $CDCl_3$, 263 K, 50 mM) and ii) The F2 projection from the ^{1}H-^{15}N HMQC experiment (500 MHz-51 MHz, $CDCl_3$, 263 K, 50 mM).

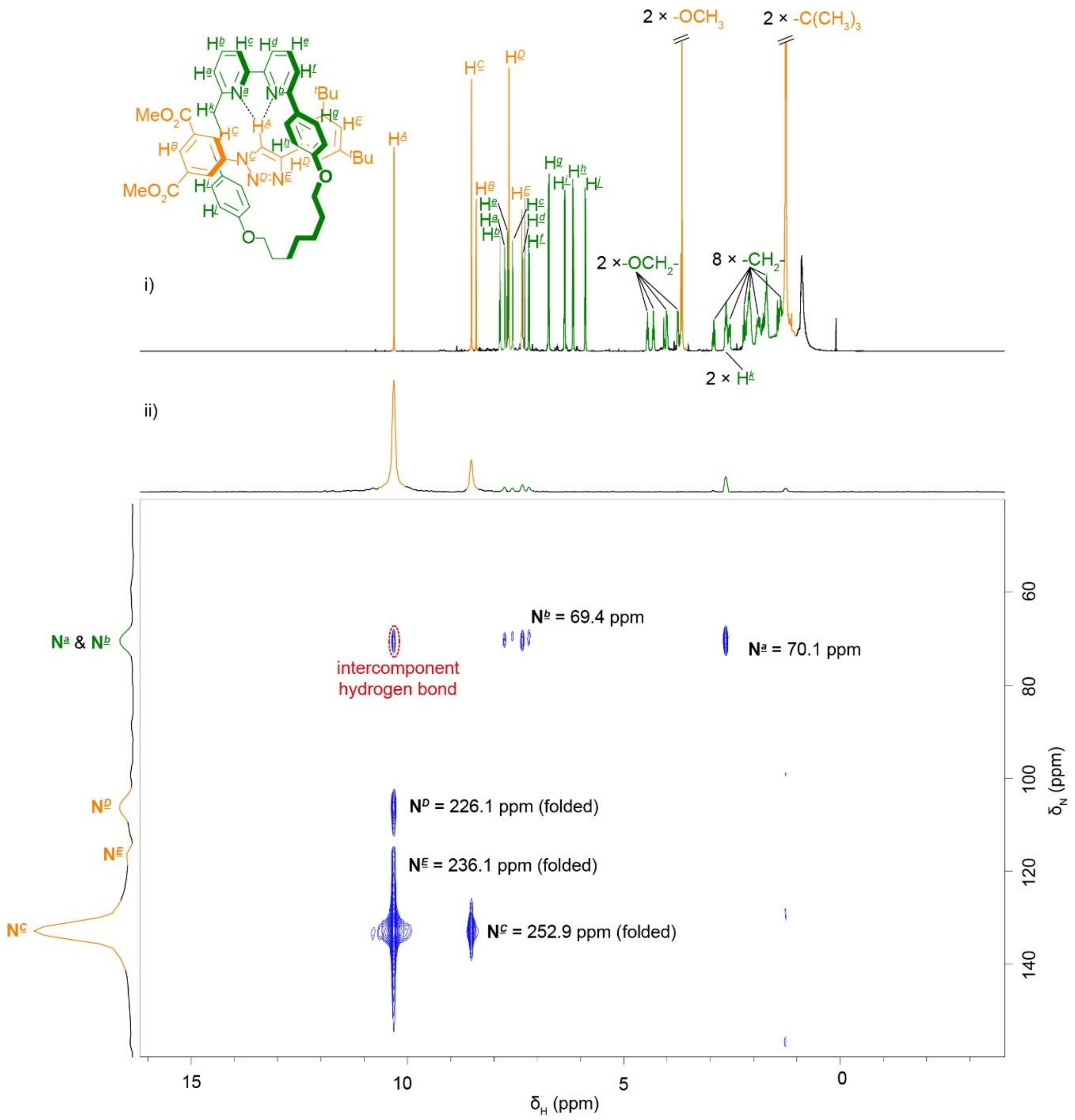

Figure S5. ^{1}H-^{15}N HMQC spectra (500 MHz-51 MHz, $CDCl_3$, 263 K, 50 mM) of [2]-rotaxane (**1**). i) The ^{1}H NMR spectra (500 MHz, $CDCl_3$, 263 K, 50 mM) and ii) The F2 projection from the ^{1}H-^{15}N HMQC experiment (500 MHz-51 MHz, $CDCl_3$, 263 K, 50 mM). The highlighted cross peak, identified by n*J* cross couplings, is indicative of intercomponent hydrogen bonds. The dotted lines indicate hydrogen bonds.

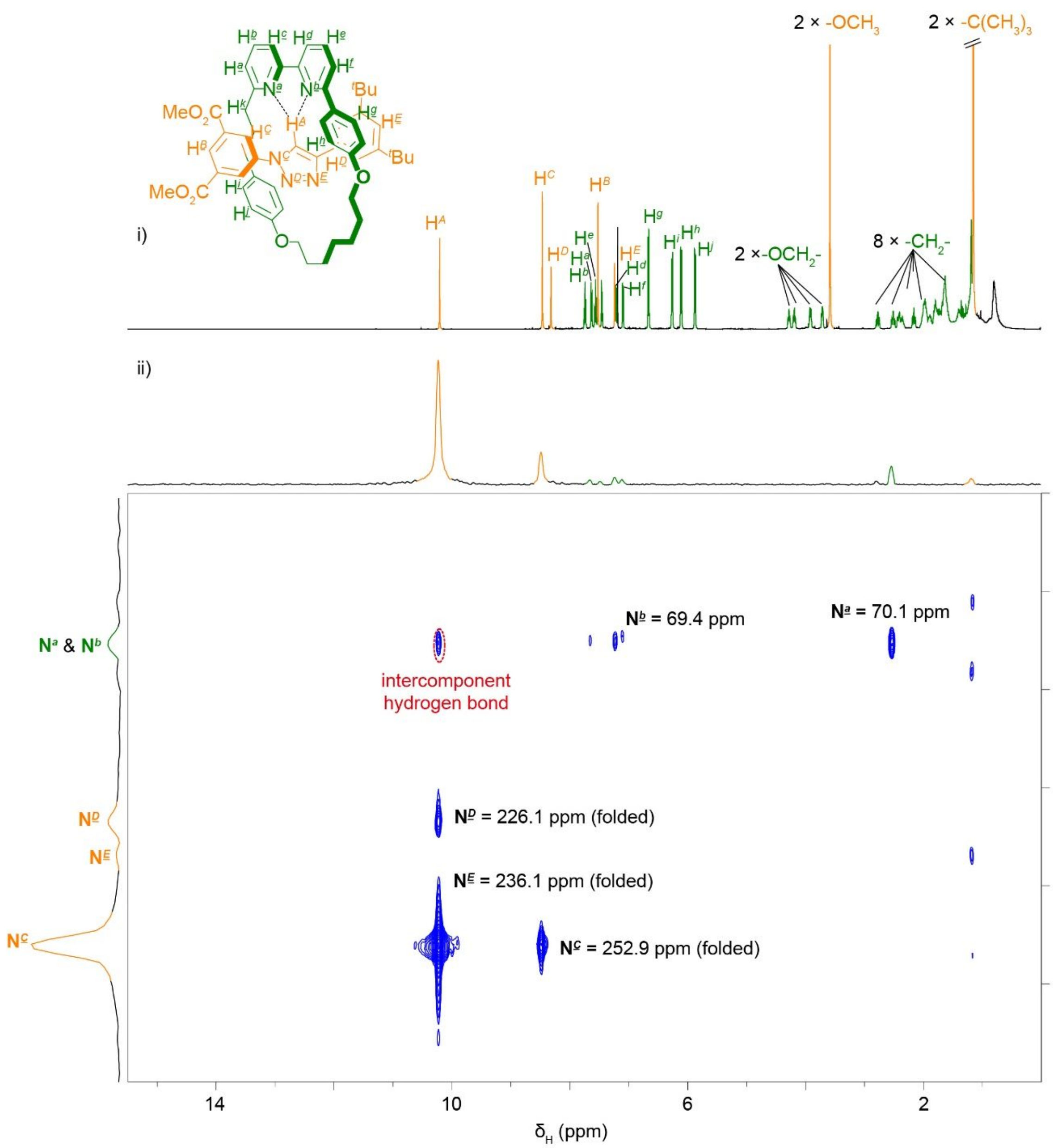

Figure S6. ^{1}H-^{15}N HMQC spectra (500 MHz-51 MHz, $CDCl_3$, 298 K, 50 mM) of [2]-rotaxane (**1**). i) The ^{1}H NMR spectra (500 MHz, $CDCl_3$, 298 K, 50 mM) and ii) The F2 projection from the ^{1}H-^{15}N HMQC experiment (500 MHz-51 MHz, $CDCl_3$, 298 K, 50 mM). The highlighted cross peak, identified by n*J* cross couplings, is indicative of intercomponent hydrogen bonds. The dotted lines indicate hydrogen bonds.

To protonate rotaxane **1** the NMR sample was diluted with chloroform (50 mL) poured onto HPF_6 (5 mL ~55 % wt aqueous solution), extracted with chloroform (2 × 50 mL), dried over magnesium sulfate, filtered and the solvent removed *in vacuo* to yield the protonated rotaxane which required no further purification (41.2 mg, quant).

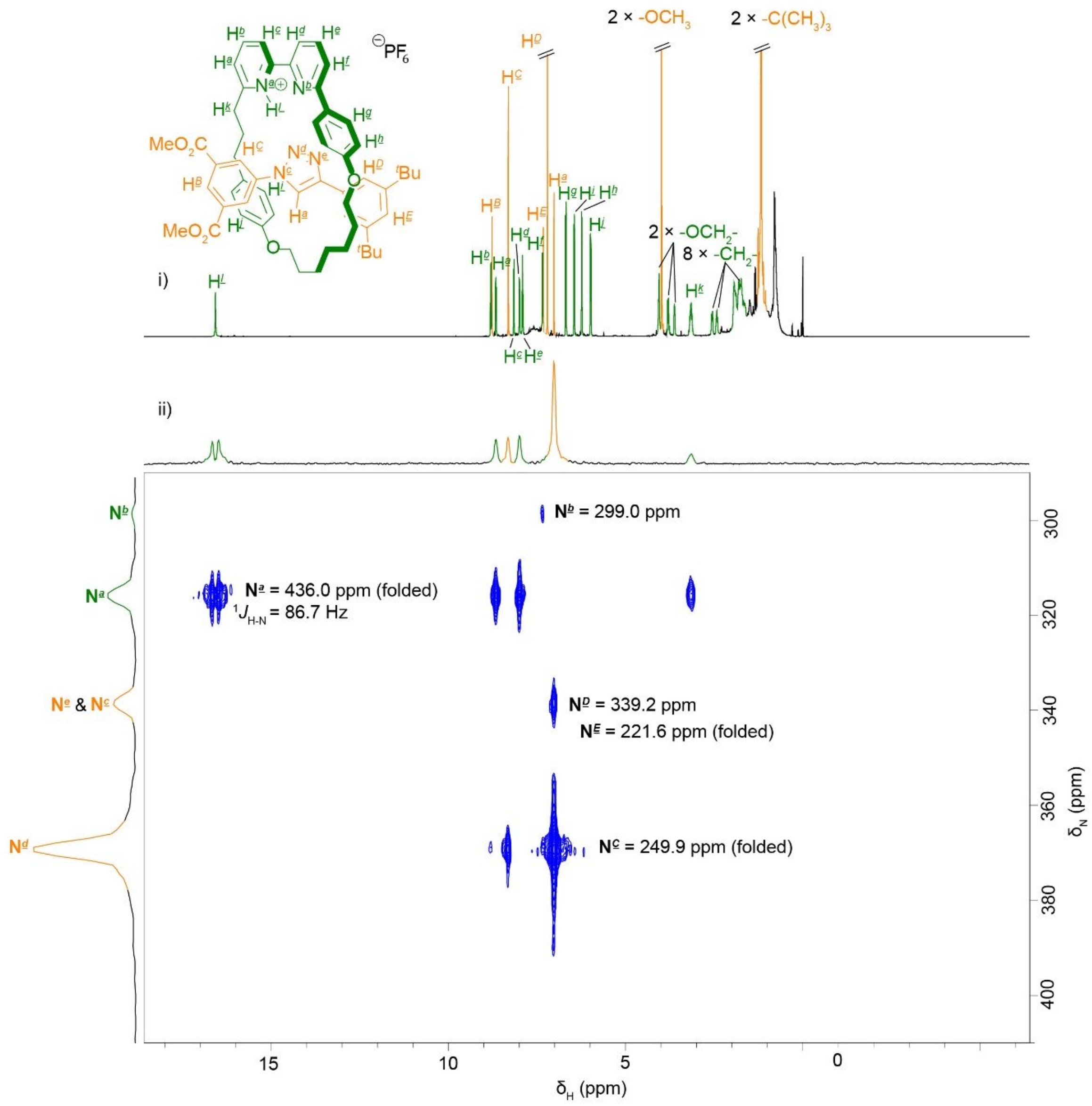

Figure S7. ^{1}H-^{15}N HMQC spectra (500 MHz-51 MHz, $CDCl_3$, 263 K, 50 mM) of protonated [2]-Rotaxane (**1**[HPF_6]). i) The ^{1}H NMR spectra (500 MHz, $CDCl_3$, 263 K, 50 mM) and ii) The F2 projection from the ^{1}H-^{15}N HMQC experiment (500 MHz-51 MHz, $CDCl_3$, 263 K, 50 mM).

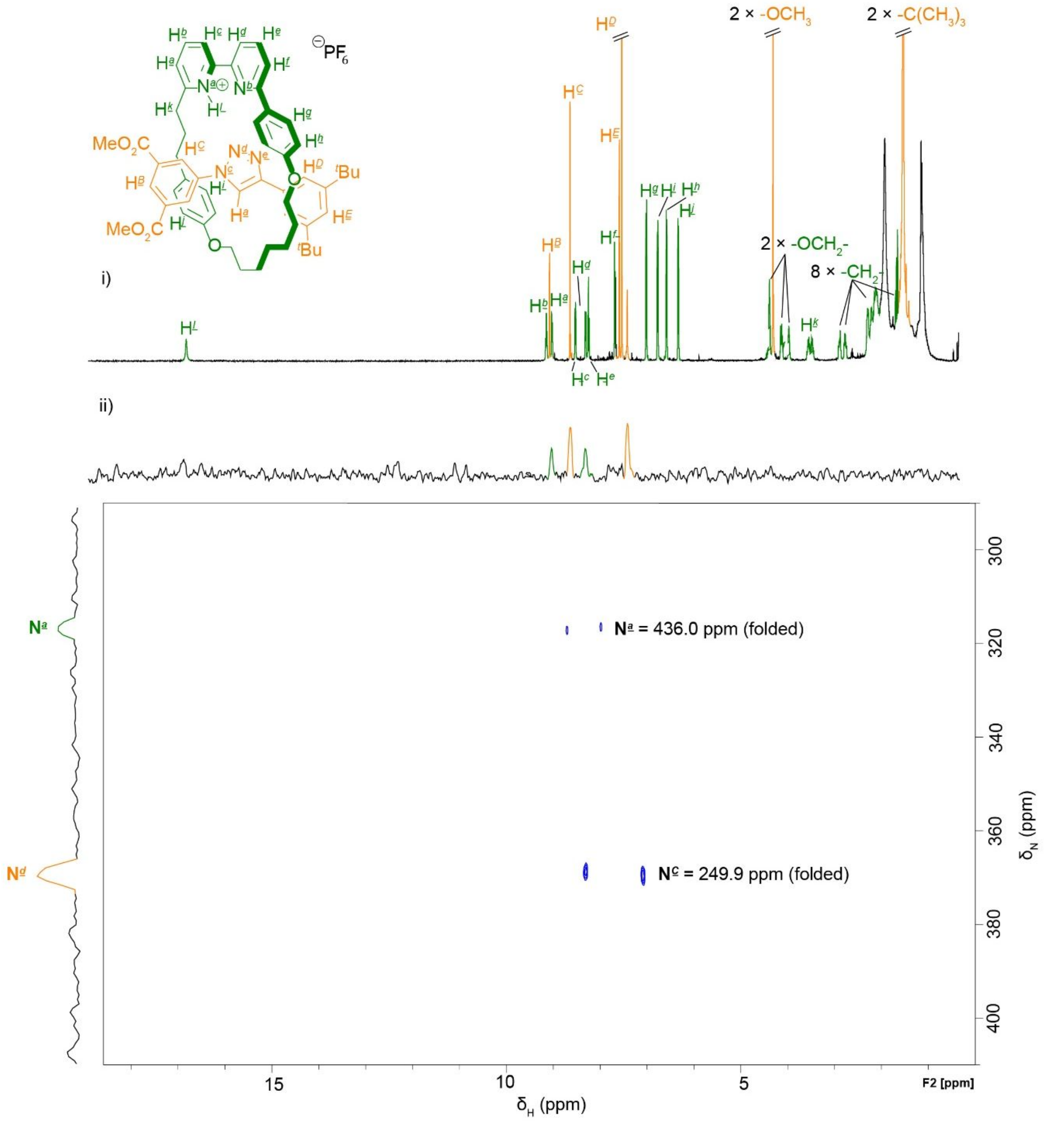

Figure S8. ^{1}H-^{15}N HMQC spectra (500 MHz-51 MHz, $CDCl_3$, 298 K, 50 mM) of protonated [2]-Rotaxane (**1**[HPF_6]). i) The ^{1}H NMR spectra (500 MHz, $CDCl_3$, 298 K, 50 mM) and ii) The F2 projection from the ^{1}H-^{15}N HMQC experiment (500 MHz-51 MHz, $CDCl_3$, 298 K, 50 mM).

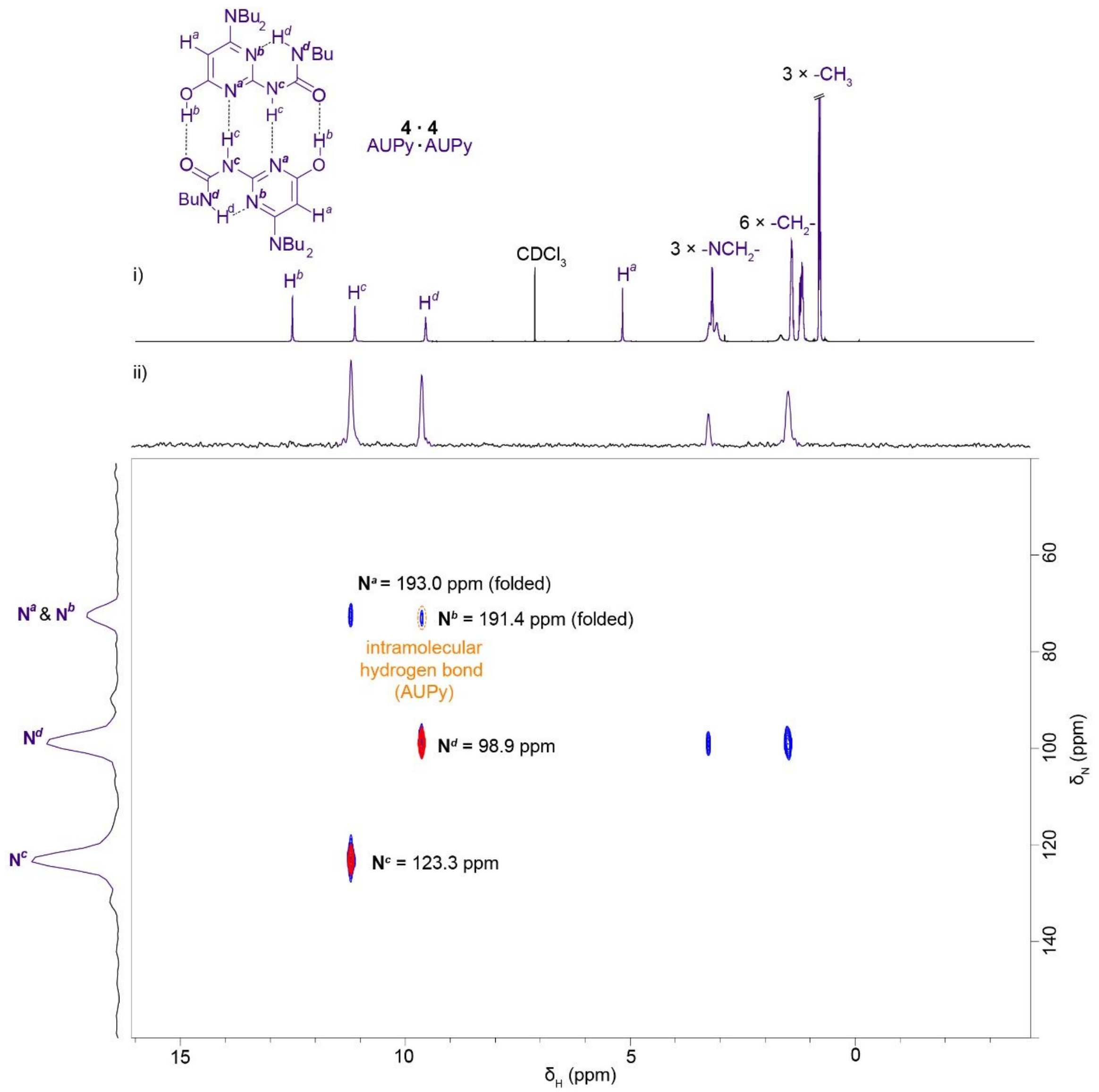

Figure S9. ^{1}H-^{15}N HMQC spectra (500 MHz-51 MHz, $CDCl_3$, 263 K, 50 mM) of AUPy▪AUPy (**4 ▪ 4**) performed on a TBO probe. i) The ^{1}H NMR spectra (500 MHz, $CDCl_3$, 263 K, 50 mM) and ii) The F2 projection from the ^{1}H-^{15}N HMQC experiment (500 MHz-51 MHz, $CDCl_3$, 263 K, 50 mM). The red cross-peaks arise from 1*J* couplings; the blue cross-peaks arise from n*J* couplings. The highlighted cross peak, identified by n*J* cross couplings, is indicative of a intramolecular hydrogen bond. The dotted lines indicate hydrogen bonds.

By changing the probe from a TBO probe to a TXI probe, all peaks were detected (**Figure S10**) including the exocyclic dibutyl amine substituent and an additional correlation between the two urea nitrogen atoms.

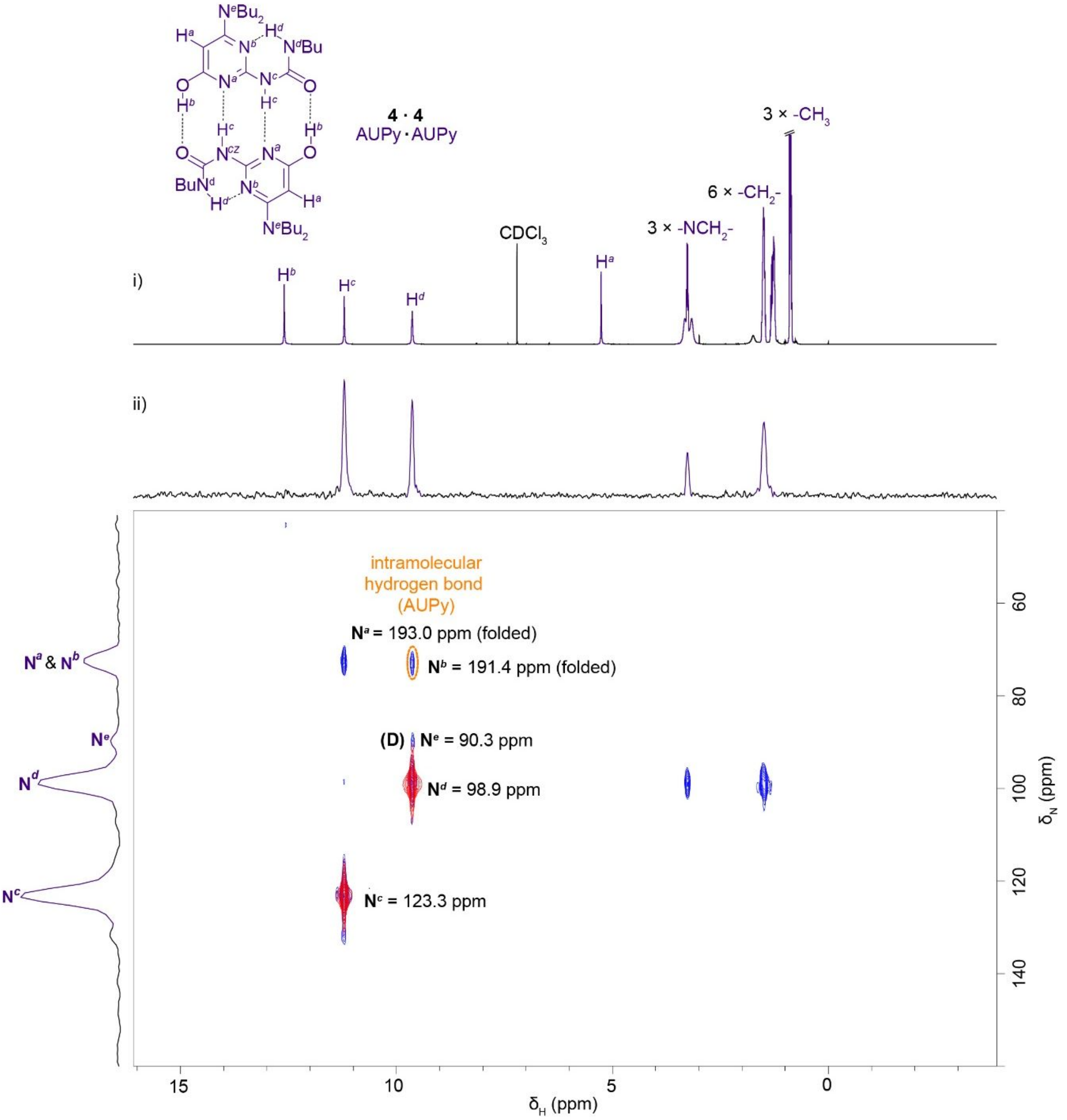

Figure S10. ^{1}H-^{15}N HMQC spectra (500 MHz-51 MHz, $CDCl_3$, 263 K, 50 mM) of AUPy▪AUPy (**4 ▪ 4**). i) The ^{1}H NMR spectra (500 MHz, $CDCl_3$, 263 K, 50 mM) and ii) The F2 projection from the ^{1}H-^{15}N HMQC experiment (500 MHz-51 MHz, $CDCl_3$, 263 K, 50 mM). The red cross-peaks arise from 1*J* couplings; the blue cross-peaks arise from n*J* couplings. The highlighted cross peak, identified by n*J* cross couplings, is indicative of a intramolecular hydrogen bond The dotted lines indicate hydrogen bonds.

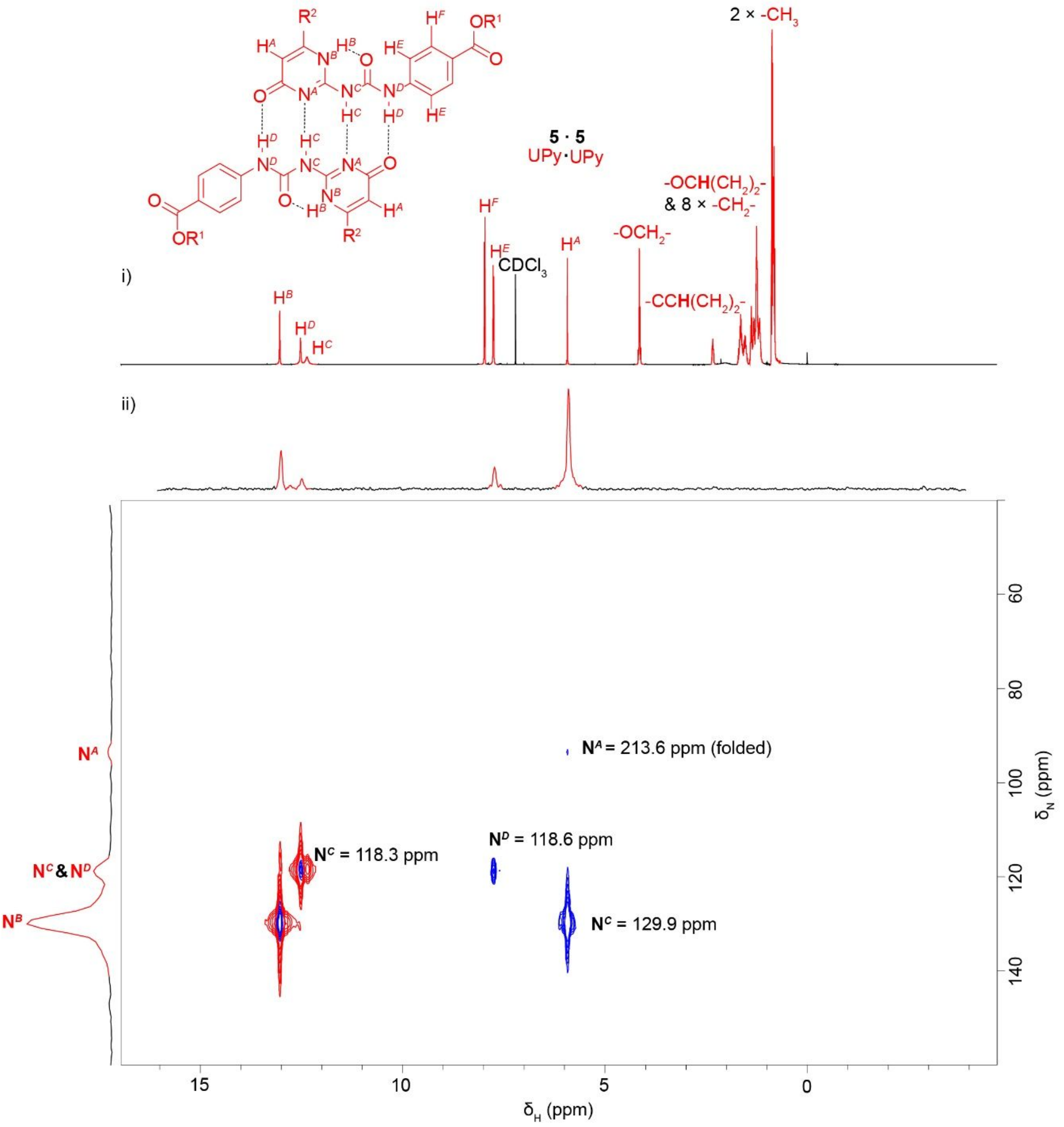

Figure S11. ^1H-^{15}N HMQC spectra (500 MHz-51 MHz, $CDCl_3$, 263 K, 50 mM) of UPy▪UPy (**5 ▪ 5**). i) The ^1H NMR spectra (500 MHz, $CDCl_3$, 263 K, 50 mM) and ii) The F2 projection from the ^1H-^{15}N HMQC experiment (500 MHz-51 MHz, $CDCl_3$, 263 K, 50 mM). The red cross-peaks arise from 1*J* couplings; the blue cross-peaks arise from n*J* couplings. The dotted lines indicate hydrogen bonds. R^1 = 2-ethylhexyl and R^2 = -CH(Et)(Bu).

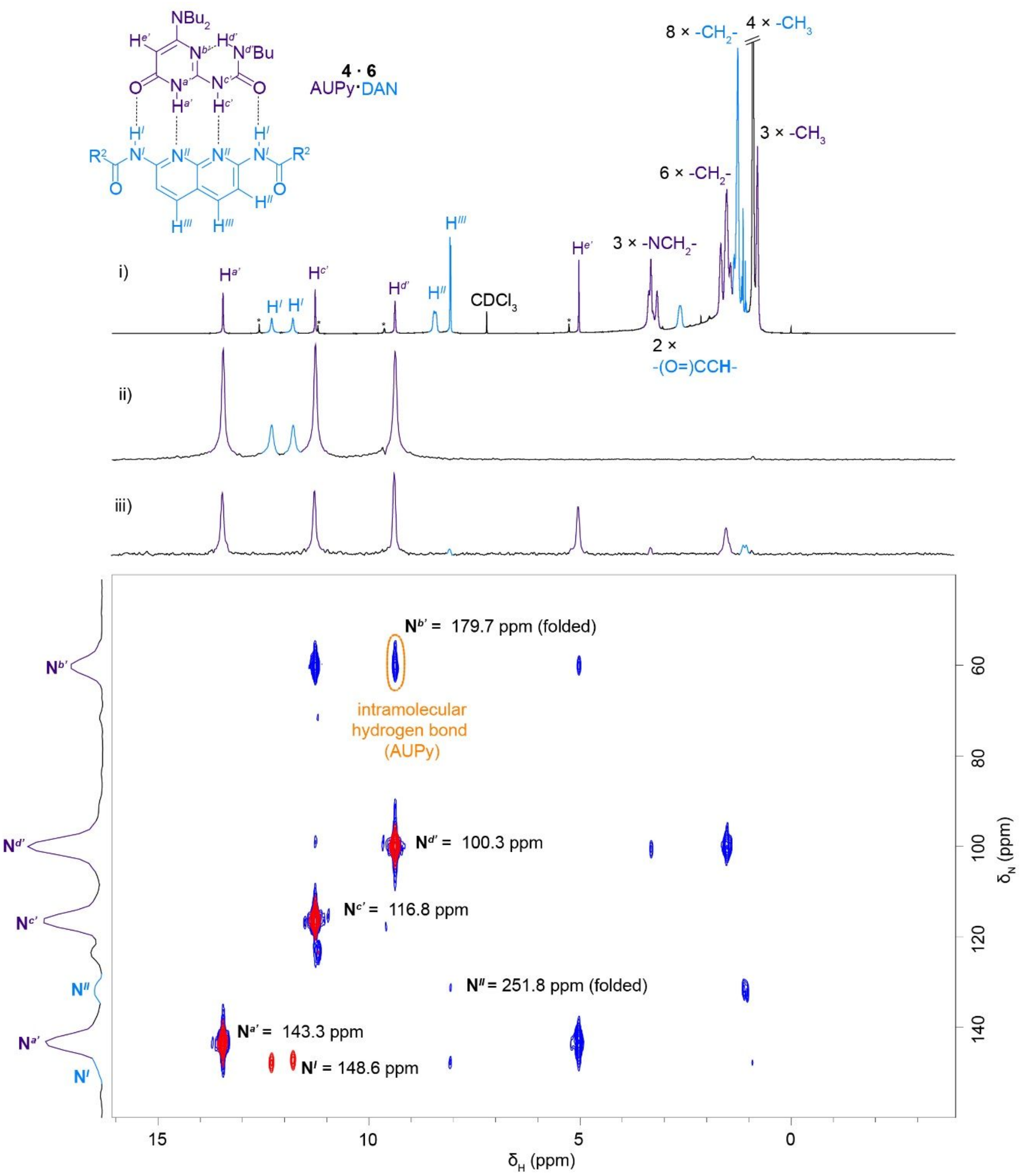

Figure S12. ^{1}H-^{15}N HMQC spectra (500 MHz-51 MHz, $CDCl_3$, 263 K, 50 mM) of ADDA▪DAAD AUPy▪DAN (**4 ▪ 6**). i) The ^{1}H NMR spectra (500 MHz, $CDCl_3$, 263 K, 50 mM), ii) The F2 projection from the 1*J* ^{1}H-^{15}N HMQC experiment (500 MHz-51 MHz, $CDCl_3$, 263 K, 50 mM) and iii) The F2 projection from the n*J* ^{1}H-^{15}N HMQC experiment (500 MHz-51 MHz, $CDCl_3$, 263 K, 50 mM). The red cross-peaks arise form 1*J* couplings; the blue cross-peaks arise from n*J* couplings. The highlighted cross peak, identified by n*J* cross couplings, is indicative of a intramolecular hydrogen bond. The dotted lines indicate hydrogen bonds. R^2 = -CH(Et)(Bu). Peaks indicated by an asterisk (*) correspond to excess AUPy (**4**).

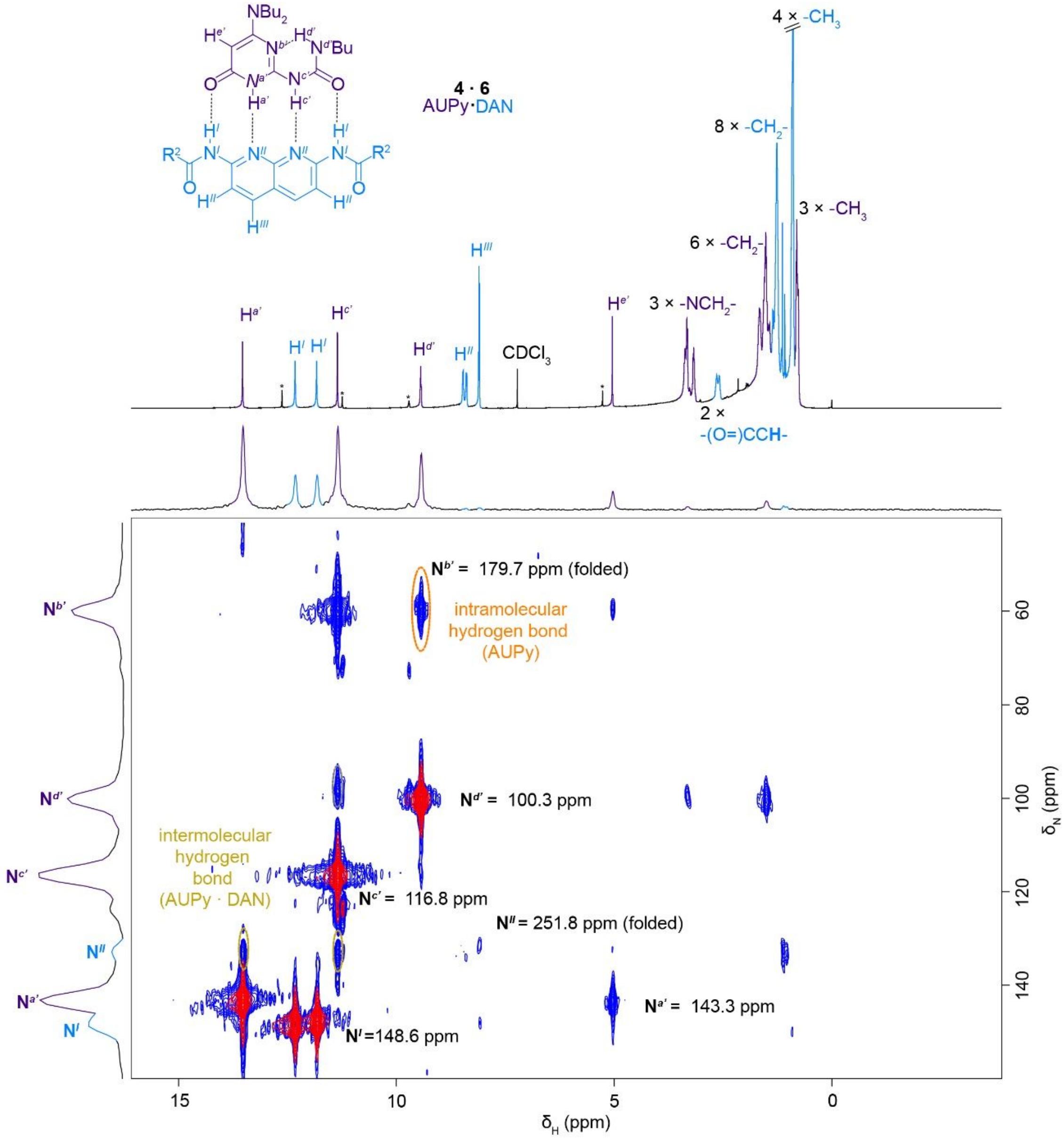

Figure S13. ^{1}H-^{15}N HMQC spectra (500 MHz-51 MHz, $CDCl_3$, 243 K, 50 mM) of ADDA▪DAAD AUPy▪DAN (**4** ▪ **6**). i) The ^{1}H NMR spectra (500 MHz, $CDCl_3$, 263 K, 50 mM), ii) The F2 projection from the 1*J* ^{1}H-^{15}N HMQC experiment (500 MHz-51 MHz, $CDCl_3$, 243 K, 50 mM) and iii) The F2 projection from the n*J* ^{1}H-^{15}N HMQC experiment (500 MHz-51 MHz, $CDCl_3$, 243 K, 50 mM). The red cross-peaks arise form 1*J* couplings; the blue cross-peaks arise from n*J* couplings. The highlighted cross peak, identified by n*J* cross couplings, are indicative of intra- and intermolecular hydrogen bonds. The dotted lines indicate hydrogen bonds. R^2 = -CH(Et)(Bu). Peaks indicated by an asterisk (*) correspond to excess AUPy (**4**).

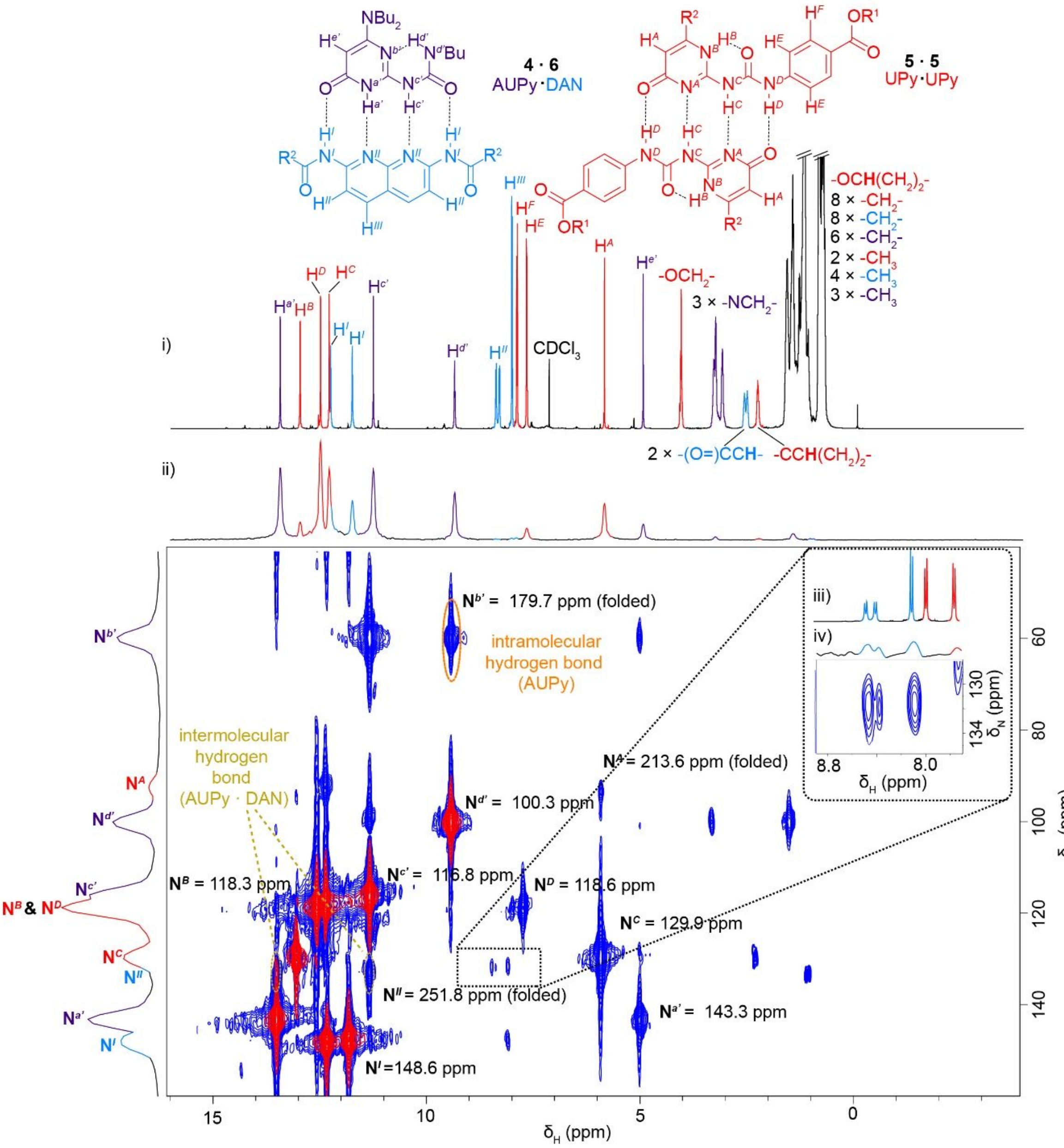

Figure S14. ^1H-^{15}N HMQC spectra (500 MHz-51 MHz, $CDCl_3$, 243 K, 50 mM) of AUPy▪DAN (**4 ▪ 6**) and UPy▪UPy (**5 ▪ 5**). i) The ^1H NMR spectra (500 MHz, $CDCl_3$, 243 K, 50 mM); ii) The F2 projection from the ^1H-^{15}N HMQC experiment (500 MHz-51 MHz, $CDCl_3$, 243 K, 50 mM); iii) Partial ^1H NMR spectra (500 MHz, $CDCl_3$, 243 K, 50 mM) for the expanded region and; iv) Partial F2 projection from the ^1H-^{15}N HMQC experiment (500 MHz-51 MHz, $CDCl_3$, 243 K, 50 mM) for the expanded region. The red cross-peaks arise form 1*J* couplings; the blue cross-peaks arise from n*J* couplings. The highlighted cross peak, identified by n*J* cross couplings, are indicative of intra- and intermolecular hydrogen bonds. The expansion indicates the relevant cross peaks showing intermolecular hydrogen bonds. The dotted lines indicate hydrogen bonds. R^1 = 2-ethylhexyl and R^2 = -CH(Et)(Bu).

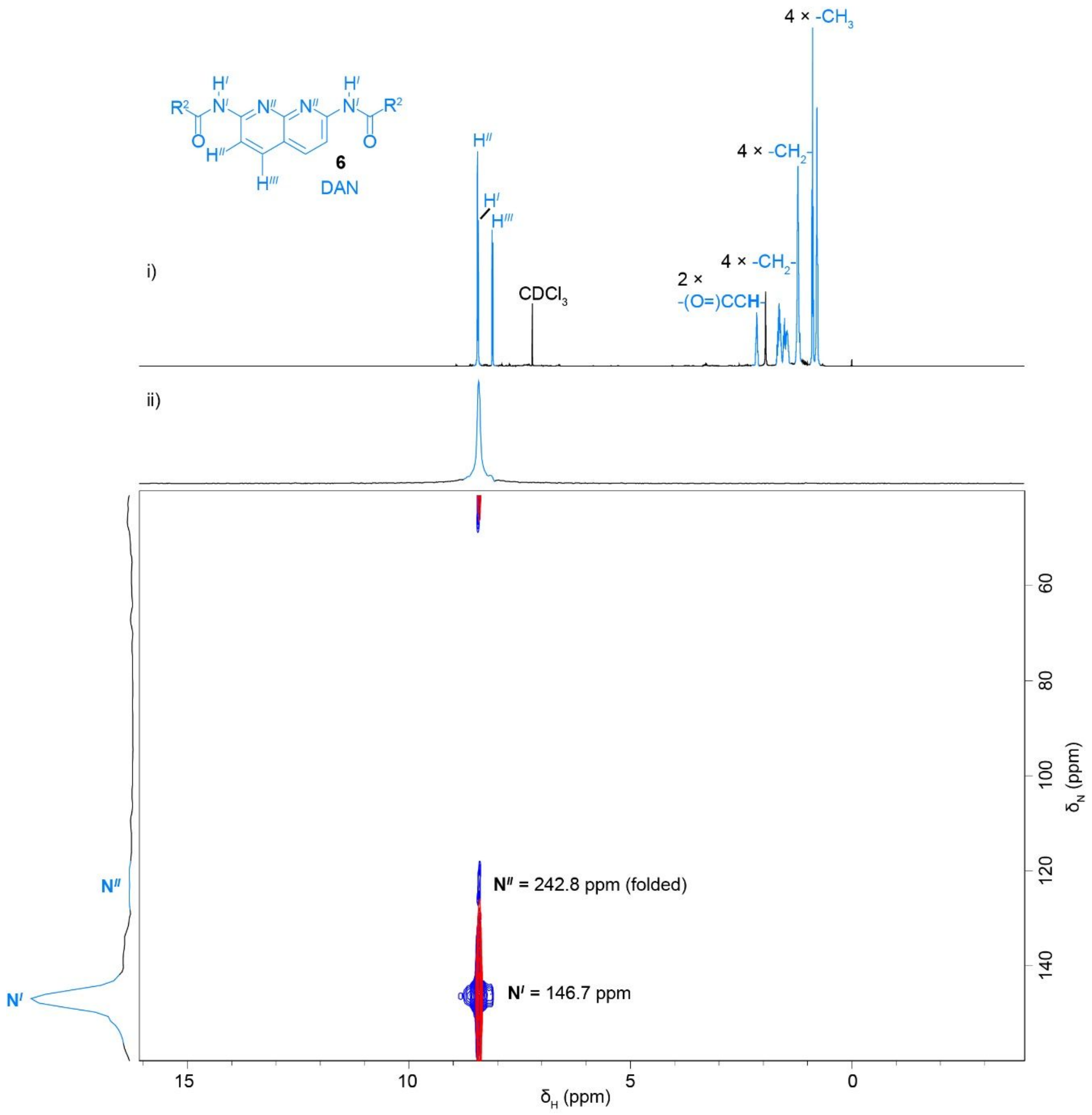

Figure S15. ^{1}H-^{15}N HMQC spectra (500 MHz-51 MHz, $CDCl_3$, 263 K, 50 mM) of DAN (**6**). i) The ^{1}H NMR spectra (500 MHz, $CDCl_3$, 263 K, 50 mM) and ii) The F2 projection from the ^{1}H-^{15}N HMQC experiment (500 MHz-51 MHz, $CDCl_3$, 263 K, 50 mM).The red cross-peaks arise form 1*J* couplings; the blue cross-peaks arise from n*J* couplings

Synthetic Procedures

General considerations: Solvents and reagents were purchased from Sigma Aldrich or Fisher Scientific and used without further purification unless otherwise stated. Where anhydrous solvents were required, dichloromethane, chloroform, tetrahydrofuran and acetonitrile were obtained from the in-house solvent purification system Innovative Inc. PureSolv®. Anhydrous pyridine was placed over KOH for 24 hours before being refluxed for 2 hours and distilled over Linde 5 Å molecular sieves and solid KOH before use. Analytical thin layer chromatography was performed on Merck Kieselgel 60 F_{254} 0.25 mm pre-coated aluminium plates. Product spots were visualised under UV light (λ_{max}=254 nm). Automated purification by flash chromatography was carried using an Isolera One (Biotage®) using 40-60 µm silica column cartridges from Biotage®. Manual flash chromatography was carried out using Merck Kieselgel 60 silica gel. Nuclear magnetic resonance spectra for all synthesized compounds, to were obtained at 298-K (unless stated otherwise) using a Bruker AV300, AV400 or AV500 spectrometer operating at 7.05 T, 9.5 T and 11.4 T (300, 400 and 500 MHz for ^{1}H respectively). Infra-red spectra were obtained using a Bruker Alfa FTIR spectrometer in which absorption maxima (ν_{max}) are expressed in wavenumbers (cm^{-1}) and only structurally relevant absorptions have been included. High-Resolution mass spectra were recorded with a BrukerDaltonics maXis Impact using electrospray ionisation (ESI) or atmospheric pressure chemical ionization (APCI).

Safety Considerations: All reactions were performed in a fume-hood, and all azides were handled with extreme care and nickel free spatulas. The temperature, when handling azides, was never allowed to exceed 303 K.

Macrocycle **3**,[1] axle components **S3**[2] and **S5**,[3] and hydrogen bonding motifs **4** (AUPy)[4] and **6** (DAN)[5] were synthesized as previously reported. Rotaxane **1** and Compound **5** (UPy), were synthesized as described below:

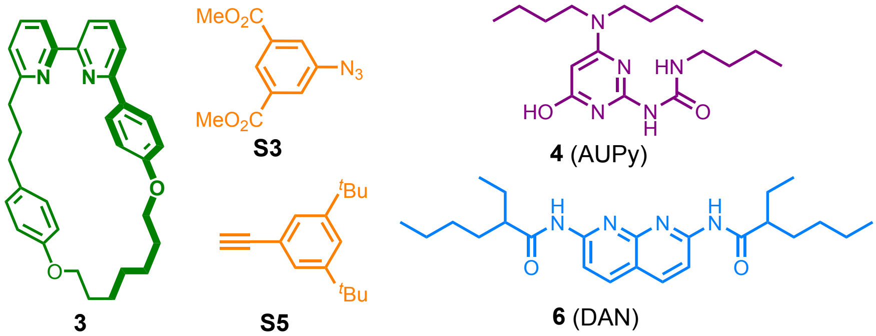

Figure S16. Literature compounds used in this study.

Novel Compound Syntheses

1 rac-R_{mp}/S_{mp}-[2]-rotaxane

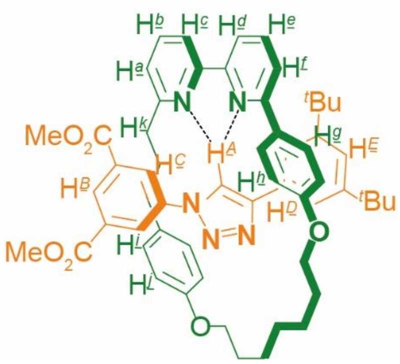

Dimethyl 5-azidoisophthalate (**S3**) (42.2 mg, 0.197 mmol), 1,3-di-*tert*-butyl-5-ethynylbenzene (**S5)** (45.3 mg, 0.196), macrocycle **3** (94.9 mg, 0.198 mmol), tetrakis(acetonitrile)copper hexafluorophosphate (72.8 mg, 0.195 mmol) and *N,N*-diisopropylethylamine (270 µL, 1.61 mmol) in dichloromethane (5 mL) was stirred at room temperature for 16 hours, protected by a nitrogen atmosphere. Trifluoroacetic acid (610 µL, 3.61 mmol) was added and the reaction was stirred for an additional 2 hours. The reaction was poured onto saturated sodium ethylenediaminetetraacetate solution (75 mL of a ~17.5 % volume aqueous ammonia solution), extracted with dichloromethane (3 × 50 mL), dried over magnesium sulfate, filtered and had the solvent removed *in vacuo.* The resultant residue was purified by automated flash chromatography (30 g, 1 : 1 40/60 petroleum ether-dichloromethane → 0 : 1 40/60 petroleum ether–dichloromethane → 9 : 1 dichloromethane–methanol) to yield the produce as a white foam (152 mg, 84 %); ^{1}H NMR (400 MHz, $CDCl_3$) δ_H 10.28 (s, 1H, H^{A}), 8.54 (d, 2H, J = 1.5 Hz, H^{C}), 8.39 (t, 1H, J = 1.6 Hz, H^{B}), 7.81 (t, 1H, J = 7.7 Hz, H^{b}), 7.70 (dd, 1H, J = 7.8 Hz, J = 1.0 Hz, H^{t}), 7.64 (t, 1H, J = 7.8 Hz, H^{e}), 7.60 (d, 2H, J = 1.8 Hz, H^{D}), 7.53 (dd, 1H, J = 7.8 Hz, J = 0.9 Hz, H^{c}), 7.31 (t, 1H, J = 1.9 Hz, H^{d}), 7.28 (dd, 1H, J = 7.7 Hz, J = 1.0 Hz, H^{a}), 7.17 (dd, 1H, J = 7.7 Hz, J = 0.9 Hz, H^{f}), 6.74 (d, 2H, J = 8.6 Hz, 2 × H^{g}), 6.34 (d, 2H, J = 8.5 Hz, 2 × H^{i}), 6.19 (d, 2H, J = 8.6 Hz, 2 × H^{h}), 5.95 (d, 2H, J = 8.5 Hz, 2 × H^{j}), 4.35 (td, 1H, J = 9.3 Hz, J = 3.2 Hz, one of -OC$\mathbf{H_aH_b}$-), 4.26 (td, 1H, J = 9.4 Hz, J = 3.5 Hz, one of -OC$\mathbf{H_aH_b}$-), 4.02-3.96 (m, 1H, one of -OC$\mathbf{H_aH_b}$-), 3.82-3.76 (m, 1H, one of OC$\mathbf{H_aH_b}$-), 3.66 (s, 6H, 2 × -OC$\mathbf{H_3}$), 2.85 (td, 1H, J = 12.8 Hz, J = 4.1 Hz, one of -OCH_2C$\mathbf{H_aH_b}$-), 2.67-2.40 (m, 3H, 2 × H^{k}, one of -OCH_2C$\mathbf{H_aH_b}$-) 2.53-2.41 (m, 1H, one of -OCH_2C$\mathbf{H_aH_b}$-), 2.13-1.62 (m, 10H, 5 × -C$\mathbf{H_2}$-), 1.51-1.44 (m, 1H, one of -OCH_2C$\mathbf{H_aH_b}$-), 1.23 (s, 18H, 2 × -C(C$\mathbf{H_3}$)$_2$); ^{13}C NMR (101 MHz, $CDCl_3$); δ_C 165.6, 163.6, 159.4, 159.3, 157.4, 157.2, 156.7, 150.7, 147.8. 137.3, 137.0, 136.7, 131.6, 131.2, 130.9, 130.2, 128.6, 128.5, 127.8, 124.4, 122.6, 122.1, 121.4, 120.0, 120.0, 119.4, 119.1, 66.7, 64.9, 51.6, 37.3, 34.9, 34.8, 34.0, 31.4, 29.4, 29.1, 27.7, 25.3, 25.2; HRMS (ESI+): calc. for $C_{58}H_{65}N_5O_6$ 928.5013 found 928.5007; FTIR (ATR): v_{max}/cm^{-1} 3065, 2951, 2925, 2859, 1731, 1605, 1567, 1512, 1462, 1449, 1433, 1245, 1175, 989.

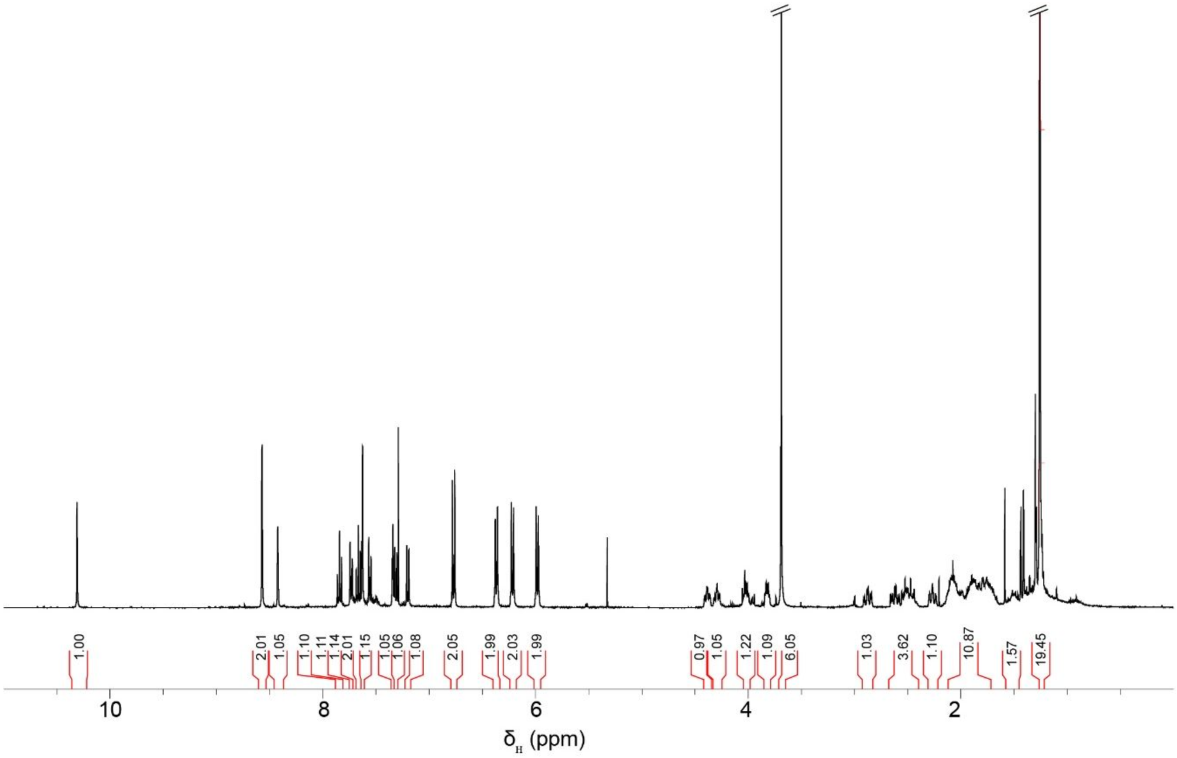

Figure S17. ^{1}H NMR (400 MHz, $CDCl_3$, 298 K).

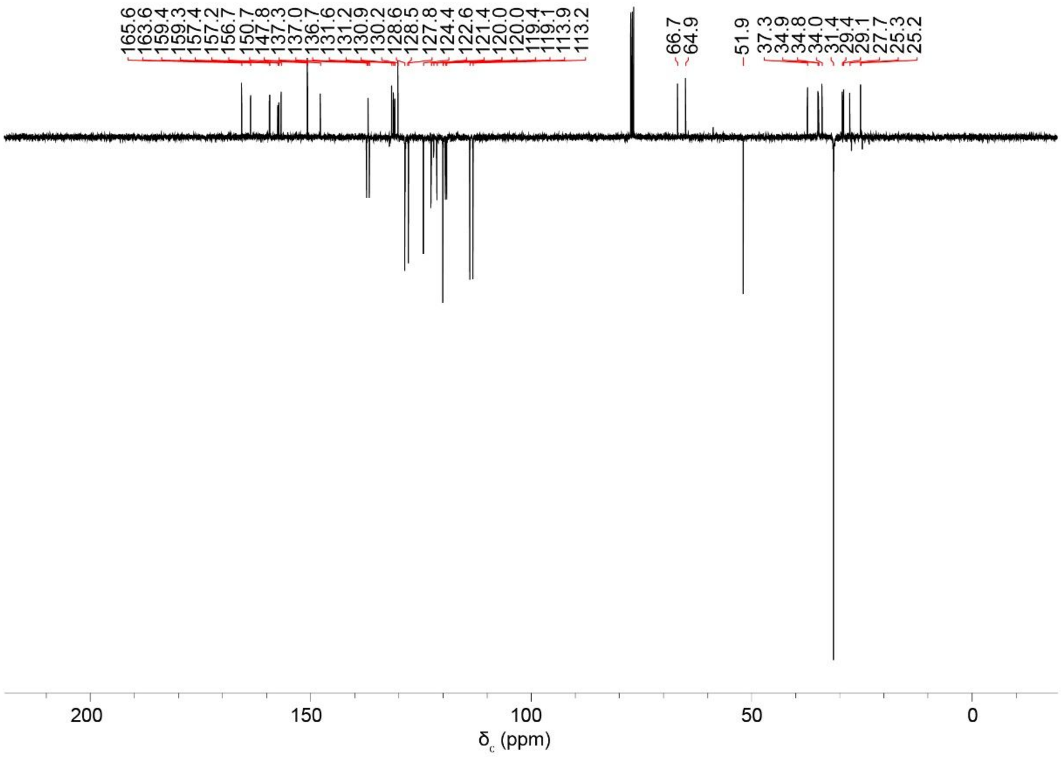

Figure S18. ^{13}C JMOD NMR (100 MHz, $CDCl_3$, 298 K).

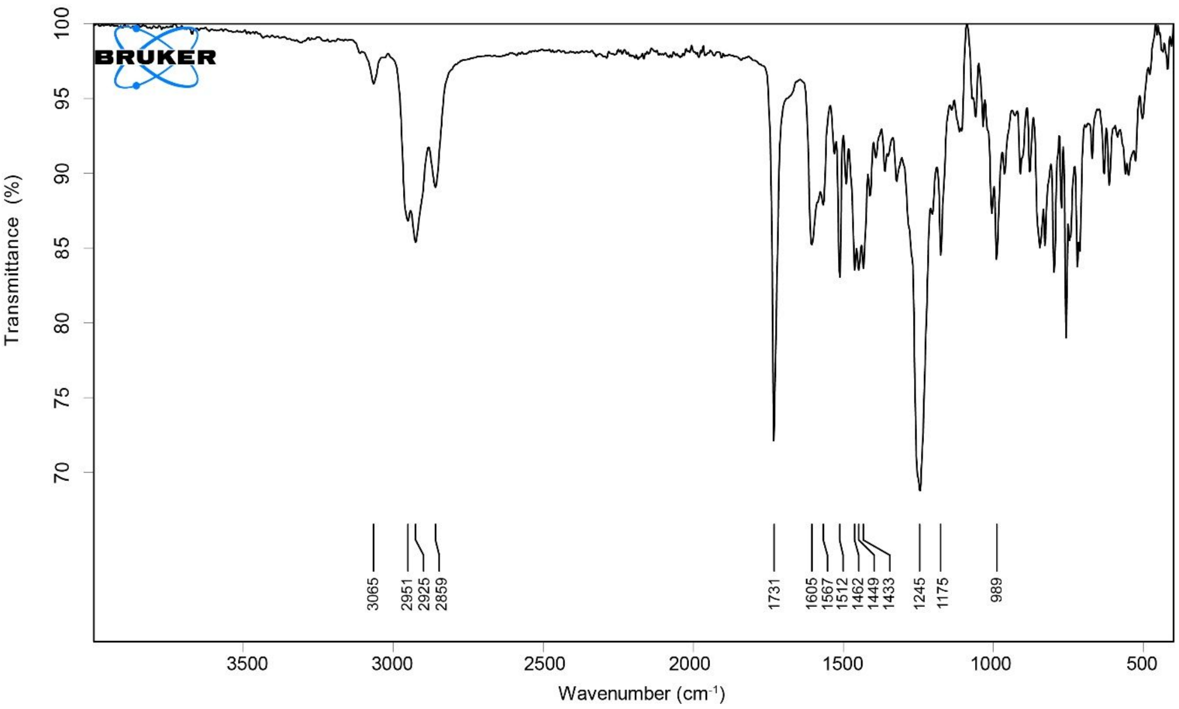

Figure S19. FTIR (ATR).

2 Dimethyl 5-(4-(3,5-di-*tert*-butylphenyl)-1*H*-1,2,3-triazol-1-yl)isophthalate

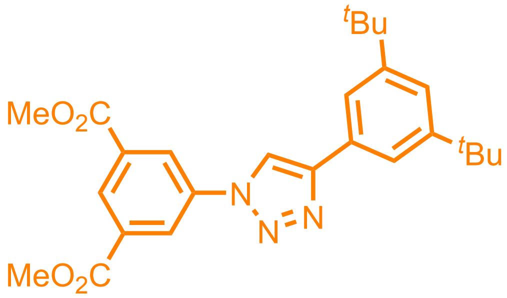

1,3-Di-*tert*-butyl-5-ethynylbenzene (**S5**) (167 mg, 0.780 mmol), dimethyl 5-azidoisophthalate (**S3**) (54.4 mg, 0.231 mmol), copper sulfate pentahydrate (5.8 mg, 0.0231 mmol) and sodium ascorbate (9.2 mg, 0.046 mmol) in dichloromethane (20 mL) and water (5 mL) was stirred at room temperature for 72 hours. The reaction was then diluted with dichloromethane (200 mL), dried over magnesium sulfate, filtered and the solvent removed *in vacuo*. The resultant residue was purified through a plug of silica (40 g silica, 1 : 1 dichloromethane-40/60 petroleum ether→100 % dichloromethane→ 9 : 1 dichloromethane-ethanol) to yield the product as an orange solid (69.1 mg, 66 %);^{1}H NMR (500 MHz, $CDCl_3$): δ_H = 8.77 (t, 1H, *J* = 1.4 Hz, Ar**H**), 8.72 (d, 2H, *J* = 1.4 Hz, 2 × Ar**H**), 8.36 (s, 1H, Triazole-**H**), 7.80 (d, 2H, *J* = 1.8 Hz, 2 × Ar**H**), 7.51 (t, 1J, *J* = 1.8 Hz, Ar**H**), 4.05 (s, 6H, 2 × -OC**H₃**), 1.43 (s, 18H, 2 × -C(C**H₃**)₃); ^{13}C NMR (126 MHz, $CDCl_3$): δ_C = 165.1, 151.6, 149.9, 137.6, 132.5, 130.2, 129.0, 125.0, 123.0, 120.4, 117.4, 52.9, 35.0, 31.5; HRMS (ESI+): calc for $C_{26}H_{31}N_3NaO_4$ 472.2202, found 472.2207; FTIR (ATR): ν_{max}/cm^{-1} 3086, 2953, 2904, 2867, 1733, 1599, 1433, 1244, 1065, 996, 756.

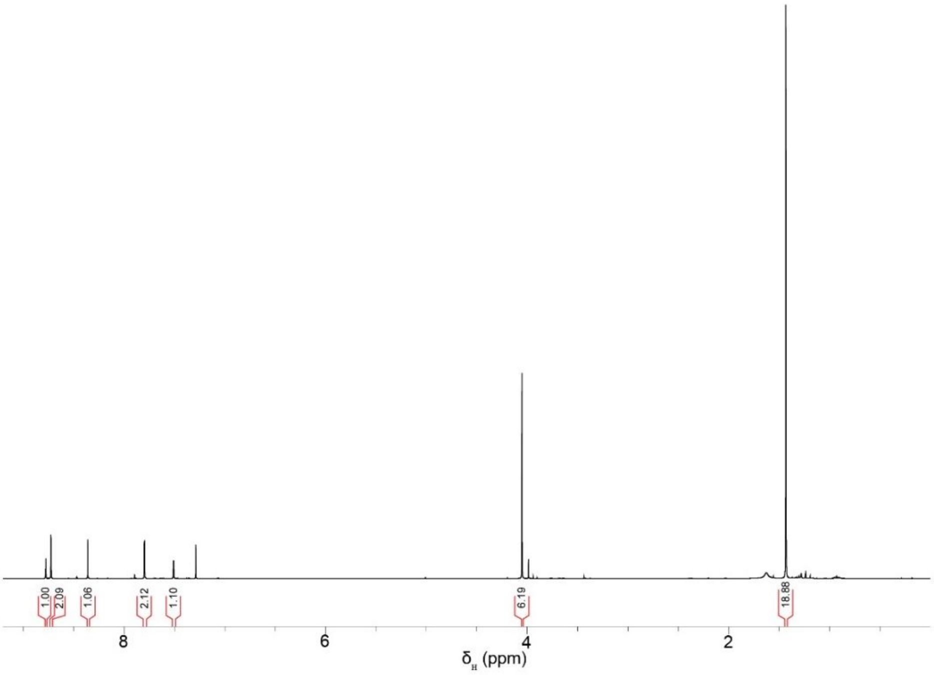

Figure S20. ^{1}H NMR (500 MHz, $CDCl_3$, 298 K).

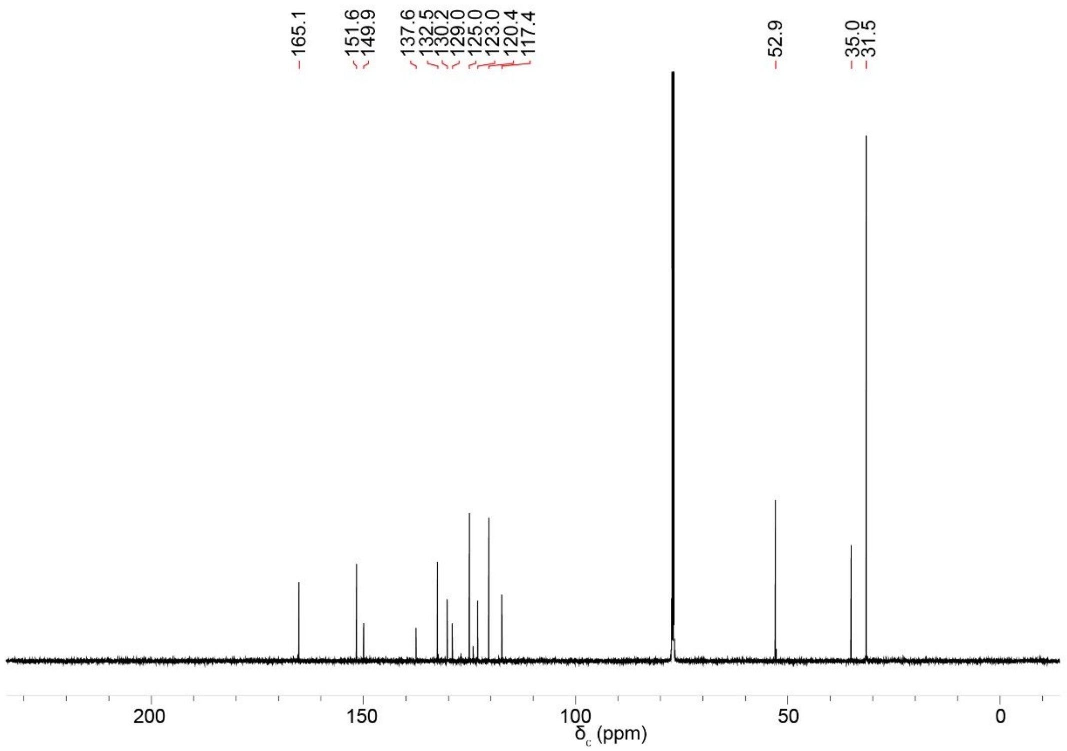

Figure S21. ^{13}C NMR (126 MHz, $CDCl_3$, 298 K).

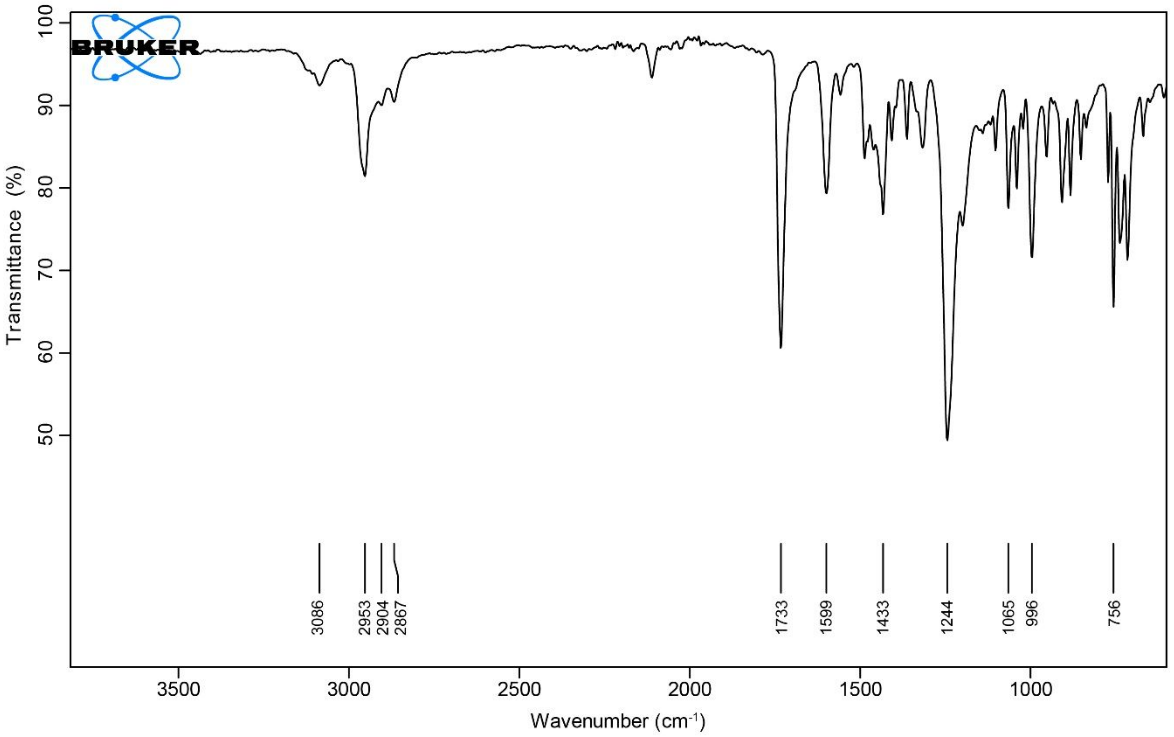

Figure S22. FTIR (ATR).

S1 2-Ethylhexyl 4-nitrobenzoate

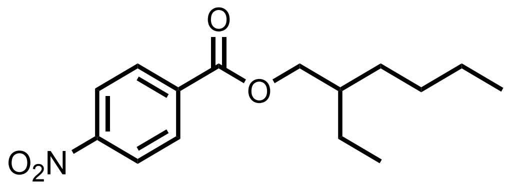

4-Nitrobenzoic acid (8.63 g, 51.6 mmol), 2-ethylhexyl bromide (11.0 mL, 62.9 mmol) potassium carbonate (14.3 g, 103 mmol) in *N,N*-dimethylformamide (100 mL) was stirred at 80 ˚C for 20 hours. The reaction was allowed to cool, poured onto water (200 mL), extracted with 40/60 petroleum ether (2 × 200 mL), washed with 5 % w/v lithium chloride (400 mL), brine (400 mL), dried with magnesium sulfate, filtered and the solvent removed *in vacuo*. The resultant oil was purified through a pad of silica (72 g, silica, 100 % 40/60 petroleum ether→100 % ethyl acetate) to yield the product as a yellow oil (12.1 g, 84 %); ^{1}H NMR (400 MHz, $CDCl_3$): δ_H = 8.22 (dt, 2H, *J* = 8.8 Hz, *J* = 2.2 Hz, 2 × Ar**H**), 8.12 (dt, 2H, *J* = 8.8 Hz, *J* = 2.2 Hz, 2 × Ar**H**), 4.22 (2 × dd, 2H, *J* = 11.1 Hz, *J* = 5.5 Hz, -OC$\mathbf{H_aH_b}$-), 1.67 (app sept., 1H, *J* = 6.3 Hz, -C**H**$(CH_2)_2$-), 1.43-1.21 (m, 8H, 4 × -C$\mathbf{H_2}$-), 0.92-0.80 (m, 6H, 2 × -C$\mathbf{H_3}$); ^{13}C NMR (100 MHz, $CDCl_3$): δ_C = 164.8, 150.5, 135.9, 130.6, 123.6, 68.3, 38.9, 30.5, 29.0, 24.0, 23.0, 14.0, 11.1; HRMS (APCI-): calc for $C_{15}H_{21}NO_4^{\bullet}$ 279.1465, found 279.1478; FTIR (ATR): v_{max}/cm^{-1} 2959, 2929, 2861, 1722, 1608, 1527, 1462, 1346, 1269, 1101, 718.

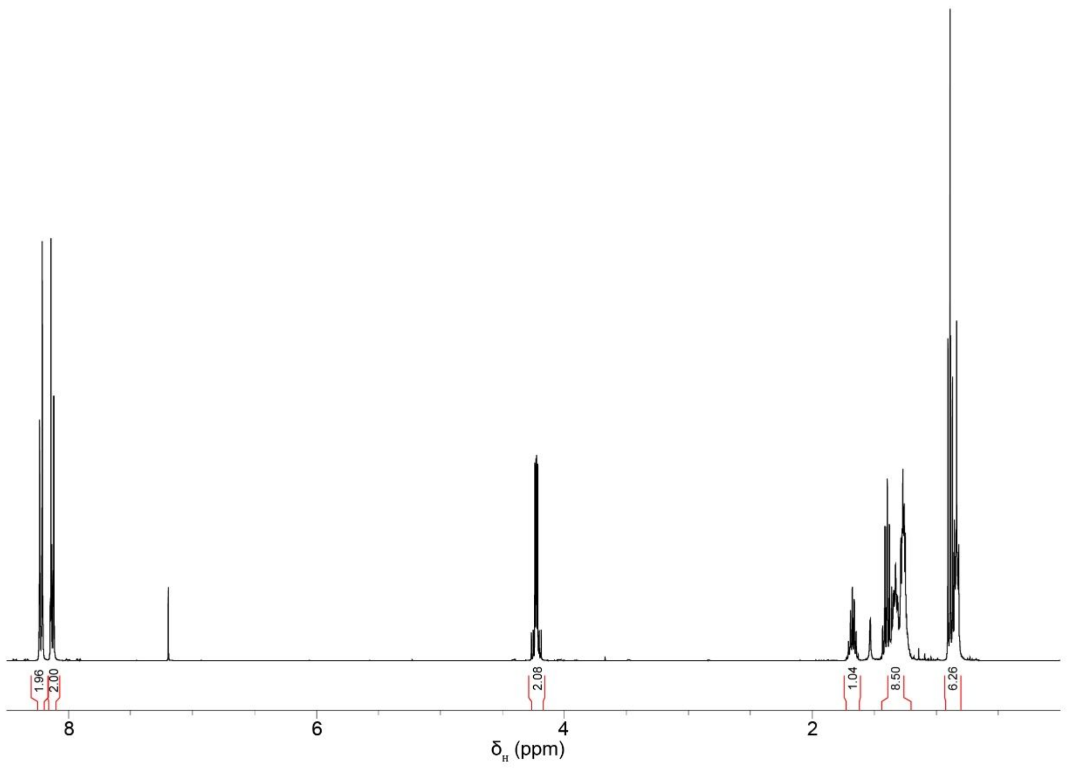

Figure S23. ^{1}H NMR (400 MHz, $CDCl_3$, 298 K).

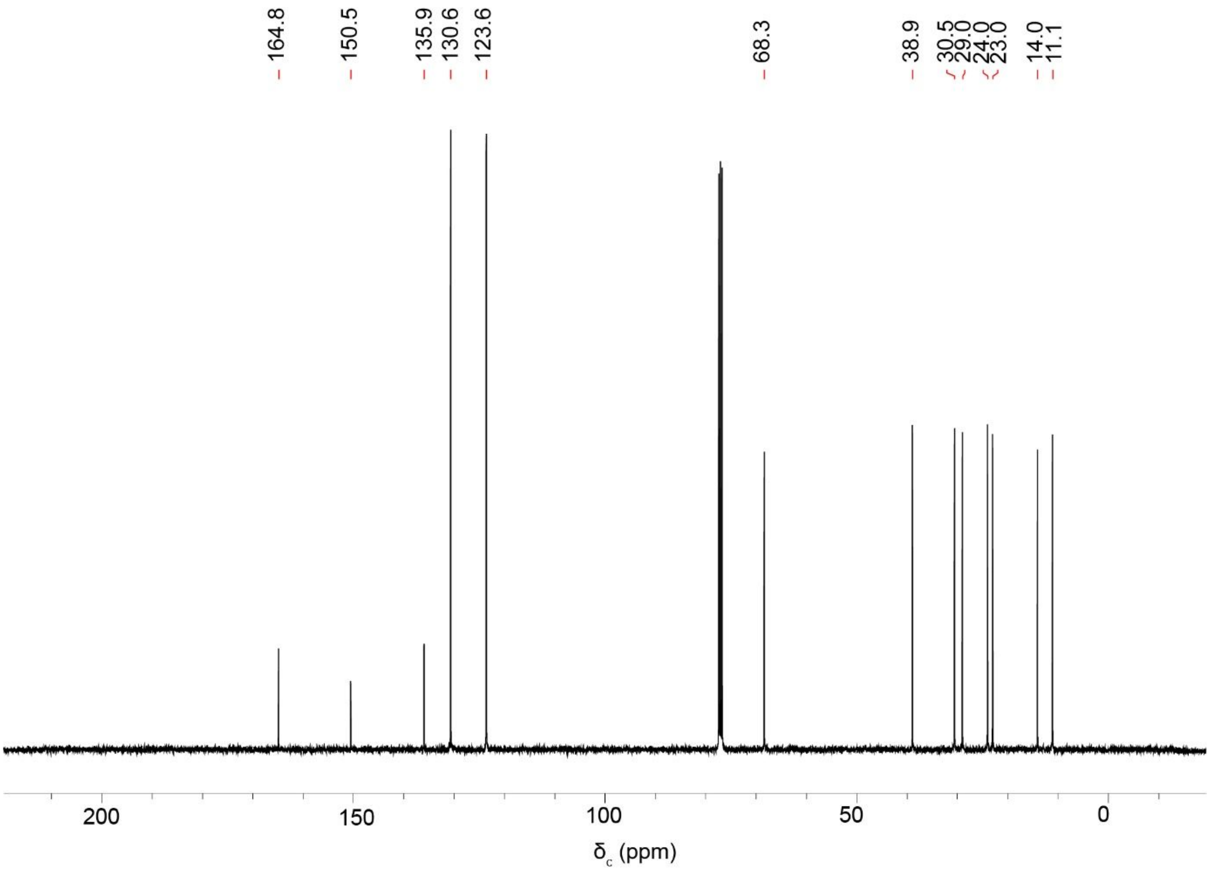

Figure S24. ^{13}C NMR (100 MHz, $CDCl_3$, 298 K).

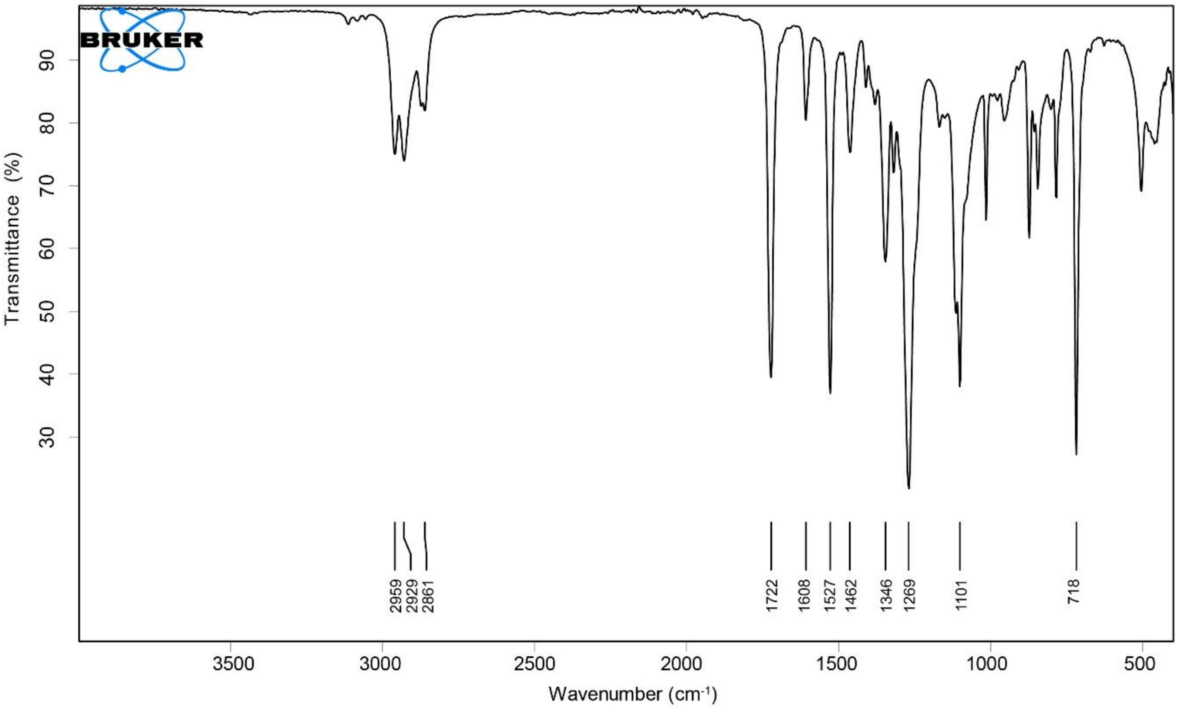

Figure S25. FTIR (ATR).

S2 2-Ethylhexyl 4-aminobenzoate

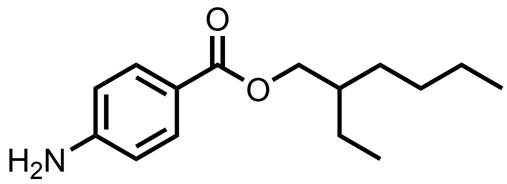

2-Ethylhexyl 4-nitrobenzoate (**S1**) (2.65 g, 9.49 mmol) and 10% wt palladium on carbon (503 mg, 0.476 mmol) in ethyl acetate (100 mL) was degassed by bubbling nitrogen through the solution for one hour. The nitrogen atmosphere was replaced with a hydrogen atmosphere and the reaction was stirred for 6 days under a hydrogen atmosphere. The hydrogen atmosphere was replaced with a nitrogen atmosphere and the reaction was then exposed to air, filtered through Celite® and the solvent removed *in vacuo* to yield the product as a yellow solid (2.27 g, 96%); ^{1}H NMR (400 MHz, $CDCl_3$): δ_H = 7.76 (dt, 2H, *J* = 8.8 Hz, *J* = 2.2 Hz, 2 × Ar**H**), 6.58 (dt, 2H, *J* = 8.8 Hz, *J* = 2.2 Hz, 2 × Ar**H**), 4.11 (2 × dd, 2H, *J* = 11.1 Hz, *J* = 5.5 Hz, -OC$\mathbf{H_aH_b}$-), 4.01-3.82 (br s, 2H, -N$\mathbf{H_2}$), 1.67 (app sept., 1H, *J* = 6.2 Hz, -C**H**$(CH_2)_2$-), 1.42-1.23 (m, 8H, 4 × -C$\mathbf{H_2}$-), 0.92-0.80 (m, 6H, 2 × -C$\mathbf{H_3}$); ^{13}C NMR (100 MHz, $CDCl_3$): δ_C = 166.9, 150.7, 131.5, 120.2, 113.8, 66.7, 39.0, 30.6, 29.0, 24.0, 23.0, 14.1, 11.1; HRMS (ESI+): calc for $C_{15}H_{24}NO_2$, 250.1802, found 250.1802; FTIR (ATR): ν_{max}/cm^{-1} 3420, 3344, 2957, 2925, 2873, 1678, 1632, 1597, 1340, 1311, 1269, 1239, 1170, 1112, 771.

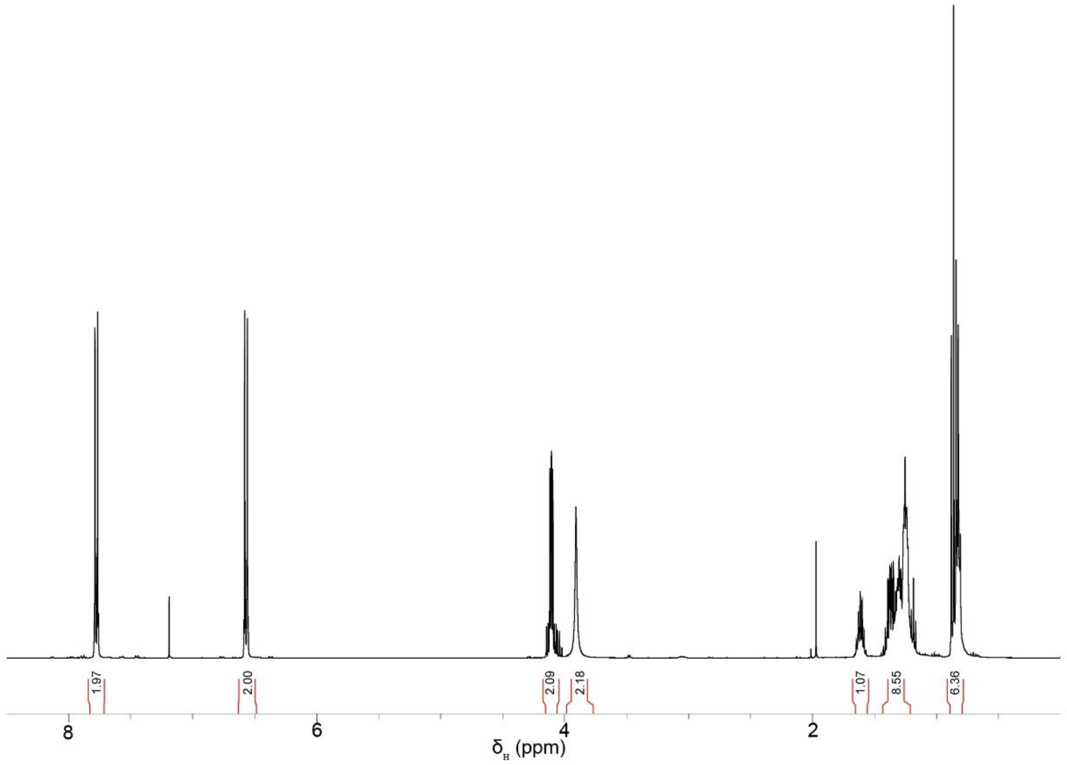

Figure S26. ^{1}H NMR (400 MHz, $CDCl_3$, 298 K).

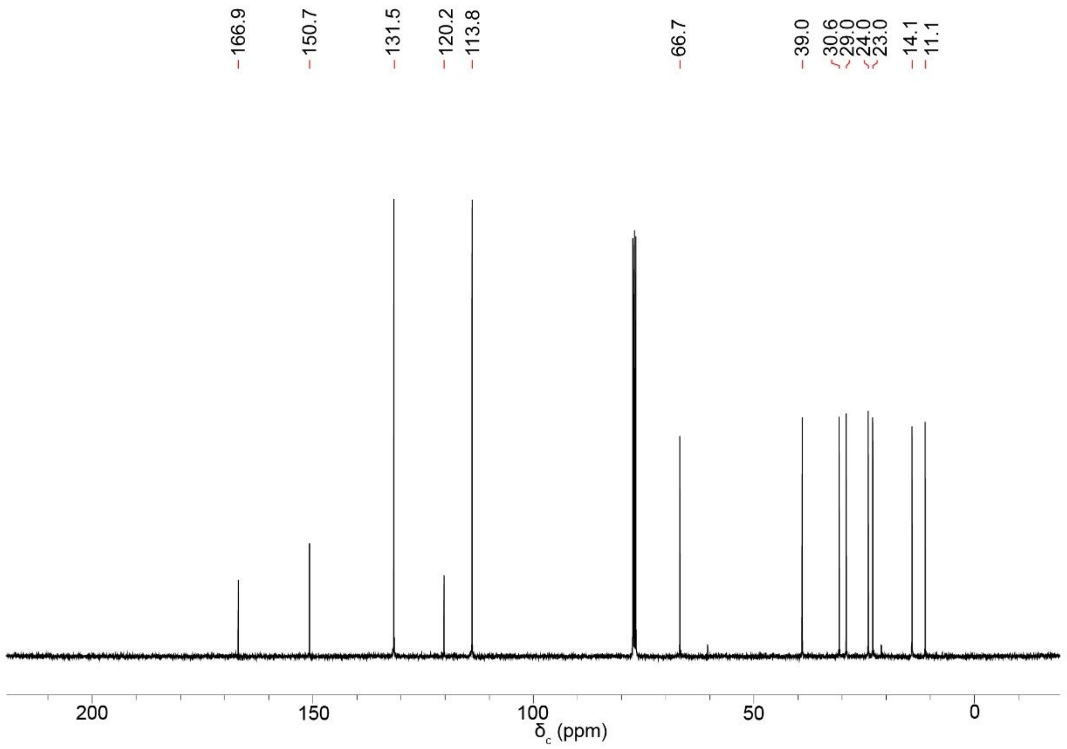

Figure S27. ^{13}C NMR (100 MHz, $CDCl_3$, 298 K).

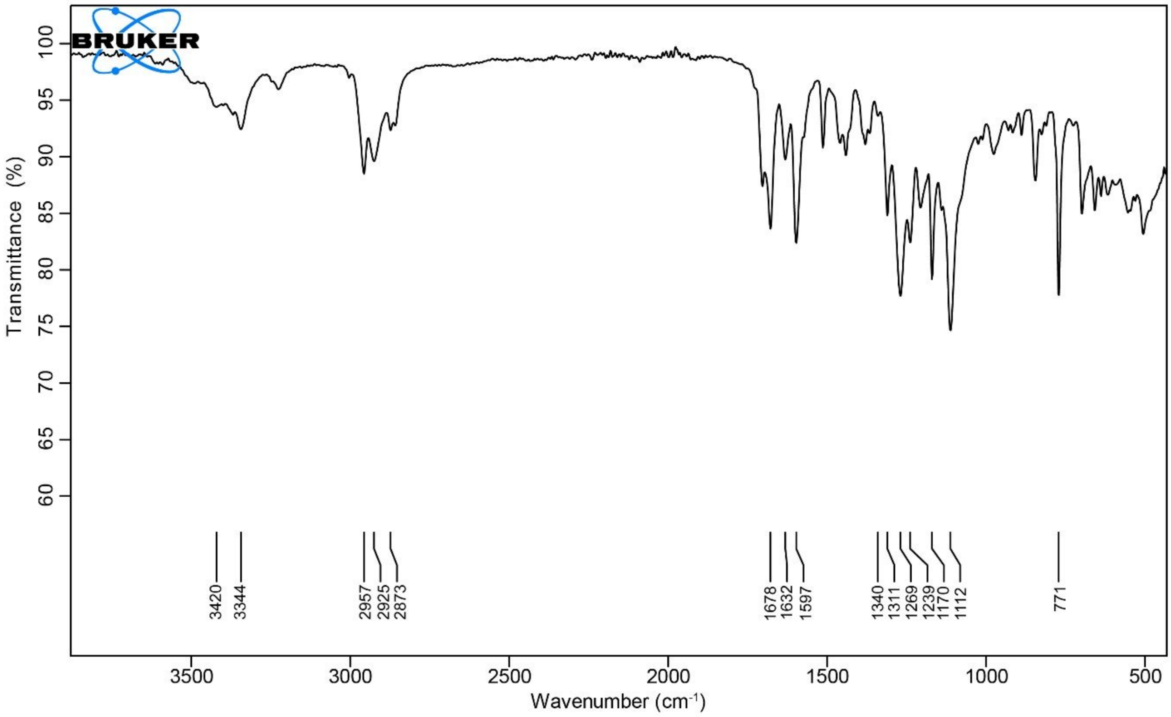

Figure S28. FTIR (ATR).

5 2-Ethylhexyl 4-(3-(6-(heptan-3-yl)-4-oxo-1,4-dihydropyrimidin-2-yl)ureido)benzoate assumed tautomer

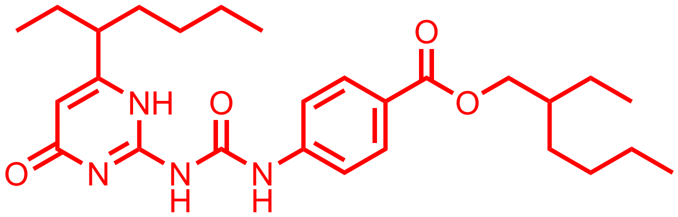

2-Amino-6-(heptan-3-yl)pyrimidin-4(1*H*)-one (**S8**) (1.24 g, 5.92 mmol) and 1,1′-carbonyldiimidazole (1.92 , 11.8 mmol) in dry chloroform (20 mL) was stirred at room temperature for 2 hours, under a nitrogen atmosphere. The reaction was diluted with chloroform (300 mL), washed with brine (4 × 200 mL), dried over magnesium sulfate, filtered and the solvent removed *in vacuo*, to yield a yellow foam (2.05 g), which contained *N*-[6-(heptan-3-yl)-4-oxo-1,4-dihydropyrimidin-2-yl]-1*H*-imidazole-1-carboxamide,[6] as indicated by ^{1}H NMR of the crude reaction mixture (**Figure S29**) which was used without further purification. 2-Ethylhexyl 4-aminobenzoate (**S2**) (704 mg, 2.83 mmol) and the *N*-[6-(heptan-3-yl)-4-oxo-1,4-dihydropyrimidin-2-yl]-1*H*-imidazole-1-carboxamide (2.05 g), *N,N*-dimethylaminopyridine (691 mg, 5.66 mmol) in dry chloroform (40 mL) was stirred at reflux for 18 h, protected by a nitrogen atmosphere. The reaction was allowed to cool, then poured onto 6 M hydrochloric acid (50 mL), extracted with dichloromethane (2 × 200 mL), dried over magnesium sulfate, filtered and the solvent removed *in vacuo*. The product was purified by crystallization from boiling acetonitrile (50 mL) as an off white solid (934 mg, 68 % with respect to 2-ethylhexyl 4-aminobenzoate); ^{1}H NMR (500 MHz, $CDCl_3$): δ_H = 13.0 (s, 1H, -N**H**), 12.64-12.11 (m, 2H, 2 × -N**H**), 7.96 (d, 2H, *J* = 9.0 Hz, 2 × Ar**H**), 7.76 (d, 2H, *J* = 9.0 Hz, 2 × Ar**H**), 5.91 (s, 1H, (O=C)C**H**-), 4.15 (dd, 2H, *J* = 11.2 Hz, *J* = 6.1 Hz, -OC**H₂**-), 2.36-2.25 (m, 1H, (O=C)C**H**$(CH_2)_2$-), 1.72-1.48 (m, 5H, -OCH_2(C**H**)-, 2 × -C**H**$_2$-), 1.43-1.16 (m, 12H, 6 × -C**H**$_2$-), 0.92-0.77 (m, 12H, 4 × -C**H**$_3$); ^{13}C NMR (126 MHz, $CDCl_3$): δ_C = 173.0, 166.5, 156.0, 154.7, 154.6, 142.5, 130.6, 125.7, 119.7, 106.8, 67.2, 42.5, 39.0, 33.0, 30.7, 29.4, 29.0, 26.7, 24.1, 23.0, 22.5, 14.1, 13.9, 11.7, 12.2; HRMS (ESI+): calc for $C_{27}H_{41}N_4O_4$, 485.3122, found 485.3120; FTIR (ATR): ν_{max}/cm^{-1} 3030, 2956, 2926, 2858, 1715, 1699, 1651, 1574, 1552, 1508, 1324, 1224, 1176, 1102, 850, 730.

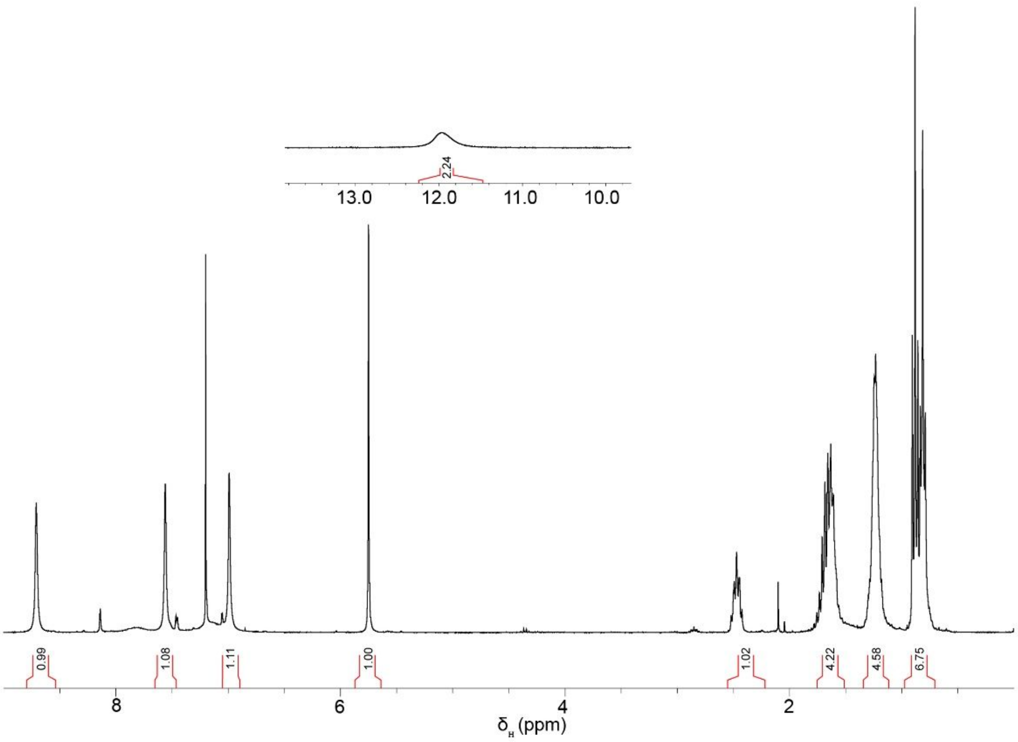

Figure S29. ^{1}H NMR (300 MHz, $CDCl_3$, 298 K) of the crude reaction mixture following reaction of 2-amino-6-(heptan-3-yl)pyrimidin-4(1*H*)-one with 1,1′-carbonyldiimidazole.

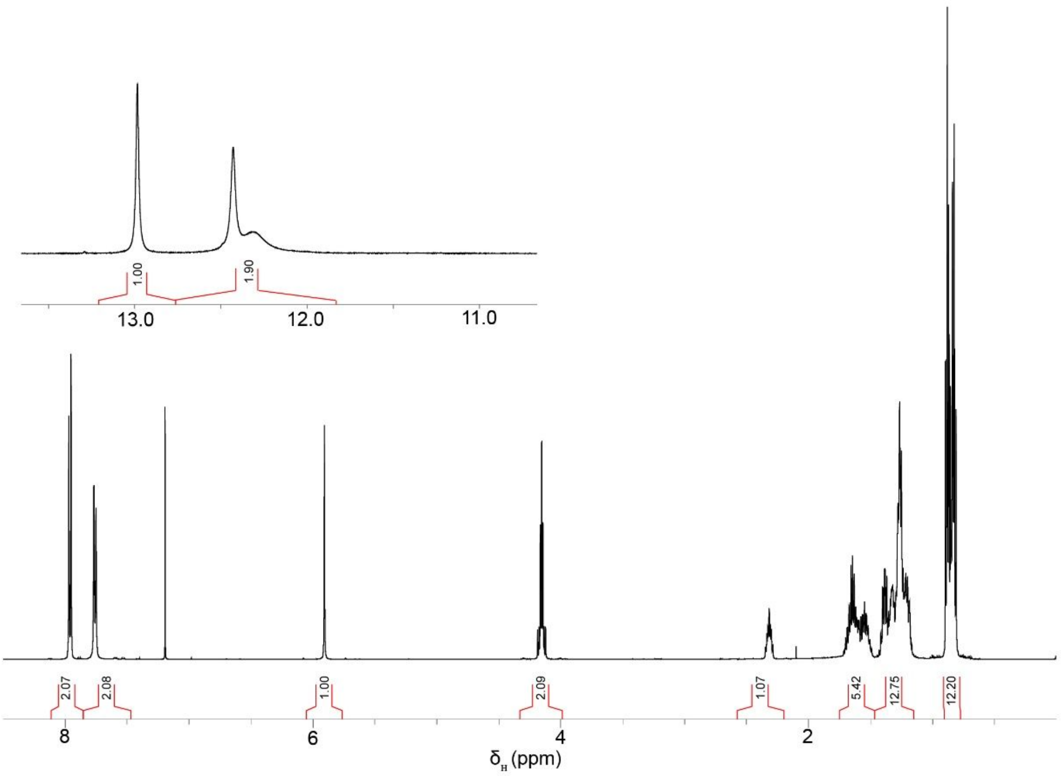

Figure S30. ^{1}H NMR (500 MHz, $CDCl_3$, 298 K)

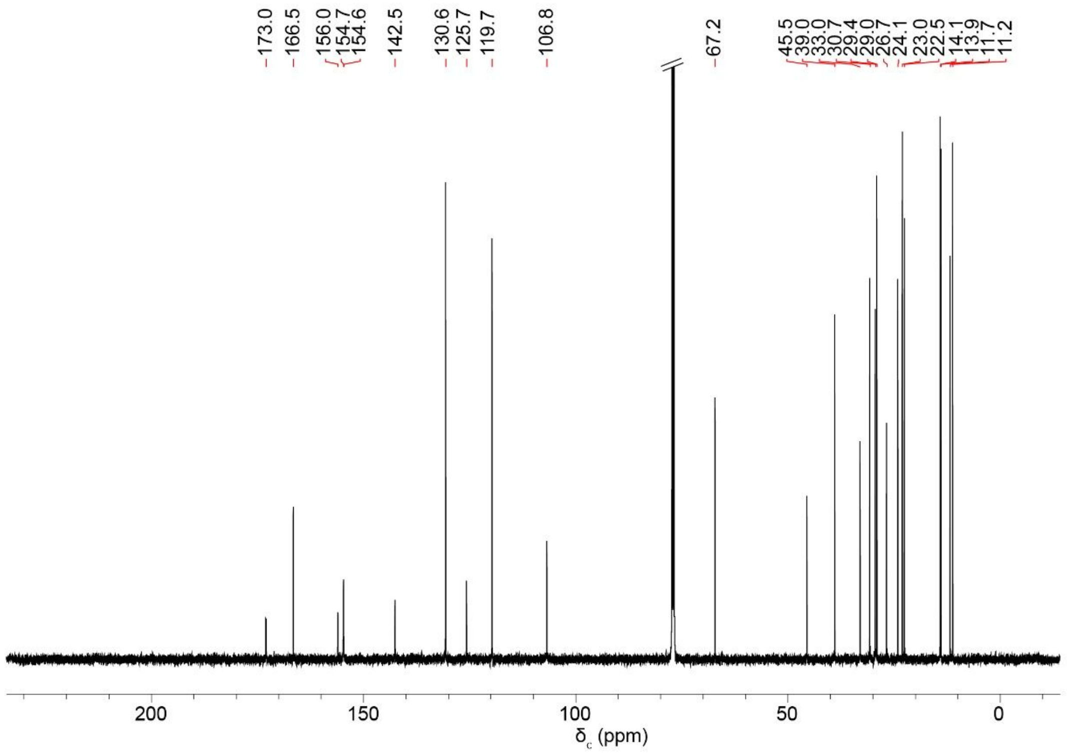

Figure S31. ^{13}C NMR (126 MHz, $CDCl_3$, 298 K).

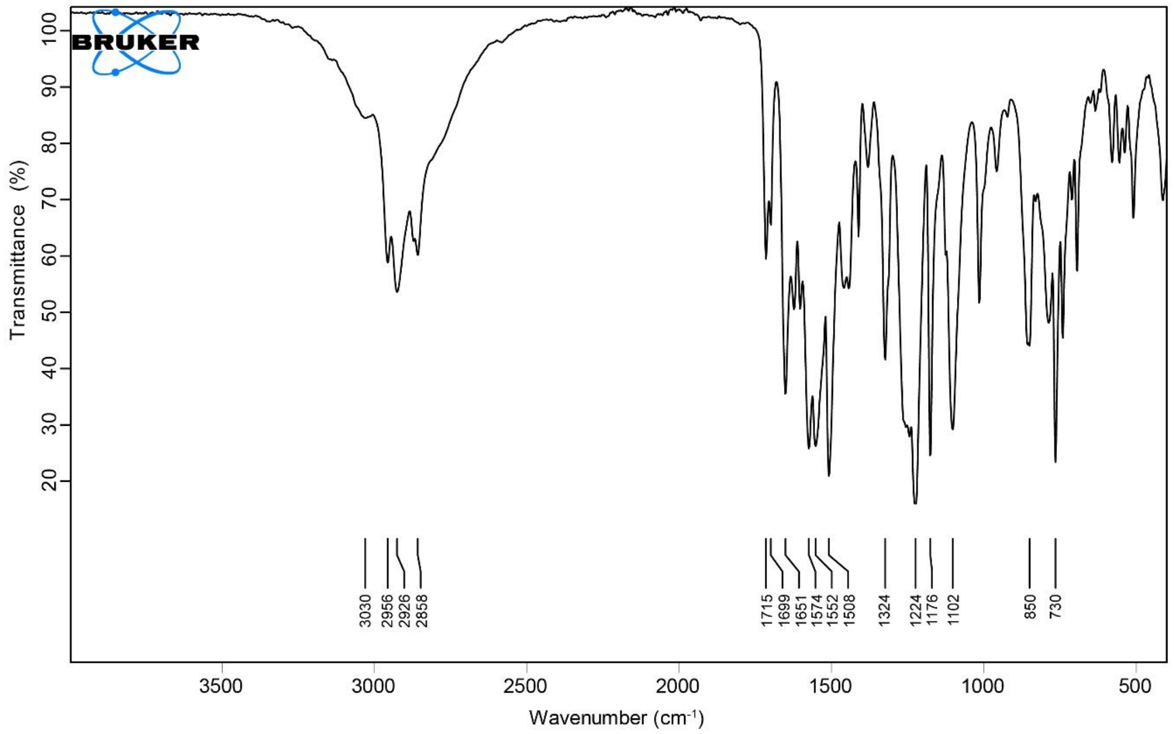

Figure S32. FTIR (ATR).

Literature Compound Syntheses

S3 Dimethyl 5-azidoisophthalate

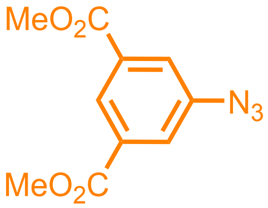

Dimethyl 5-aminoisophthalate (744 mg, 3.56 mmol) in aqueous hydrochloric acid (~37 % w/v, 20 mL) and deionized water (20 mL) was cooled to 0 °C. Sodium nitrite (258 mg, 3.74 mmol) in deionized water (5 mL) was added and the reaction was stirred at 0 °C for 30 minutes, then exposed to air. Sodium azide (243 mg, 3.74 mmol) in deionized water (5 mL) was added and the reaction was stirred at 0 °C with warming to room temperature for 21 hours, then exposed to air. The precipitate was filtered off, and washed with deionized water (500 mL), then dissolved in dichloromethane (200 mL), dried over magnesium sulfate, filtered, and the solvent carefully removed *in vacuo*, to yield a yellow solid which required no further purification (546 mg, 65 %); ^{1}H NMR (300 MHz, $CDCl_3$): δ_H = 8.45 (t, 1H, *J* = 1.5 Hz, Ar**H**), 7.88 (d, 2H, *J* = 1.5 Hz, 2 × Ar**H**), 3.98 (s, 6H, 2 × -C**H₃**). The data is in agreement with the literature.[2]

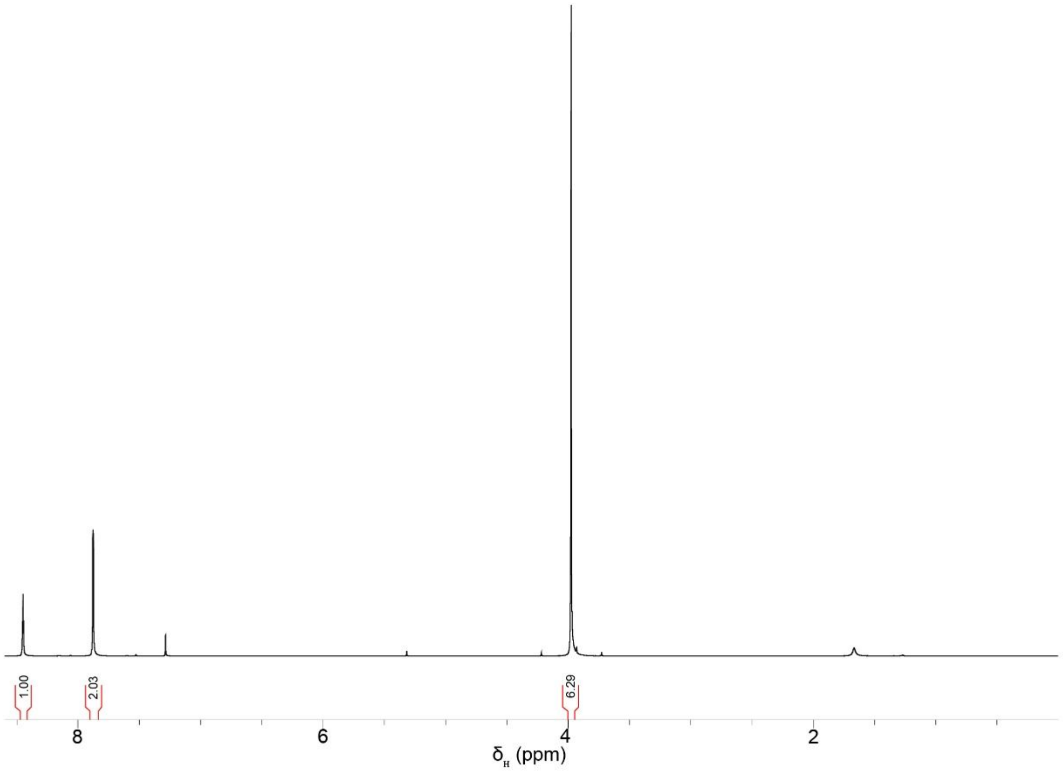

Figure S33. ^{1}H NMR (300 MHz, $CDCl_3$, 298 K).

S4 ((3,5-Di-*tert*-butylphenyl)ethynyl)trimethylsilane

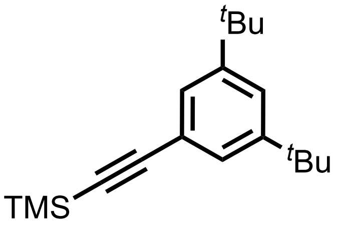

In a microwave vial with PTFE septa, 1-Bromo-3,5-di-*tert*-butylbenzene (17.47 g, 61.5 mmol), bis(triphenylphosphine)palladium(II) dichloride (1.53 g, 2.18 mmol), and copper(I) iodide (913 mg, 4.79 mmol) in triethylamine (30 mL) was degassed by bubbling nitrogen through the suspension for 30 minutes. Ethynyltrimethylsilane (16 mL, 123 mmol) was added and the reaction was stirred at 70 °C for 16 hours. The reaction was allowed to cool, the solvent removed *in vacuo*, and the resultant residue was partially purified through a plug of silica (50 g SiO_2, solid load, 100 % 40/60 petroleum ether) to yield the product as a white solid (17.48 g, 99 %), which was used without further purification; ^{1}H NMR (400 MHz, $CDCl_3$): δ_H = 7.40 (t, 1H, *J* = 2.0 Hz, Ar**H**), 7.34 (s, 2H, *J* = 2.0 Hz, 2 × Ar**H**), 1.34 (s, 18H, 2 × -C(C**H₃**)₃), 0.29 (s, 9H, -Si(C**H₃**)₃). The data is in agreement with the literature.[3]

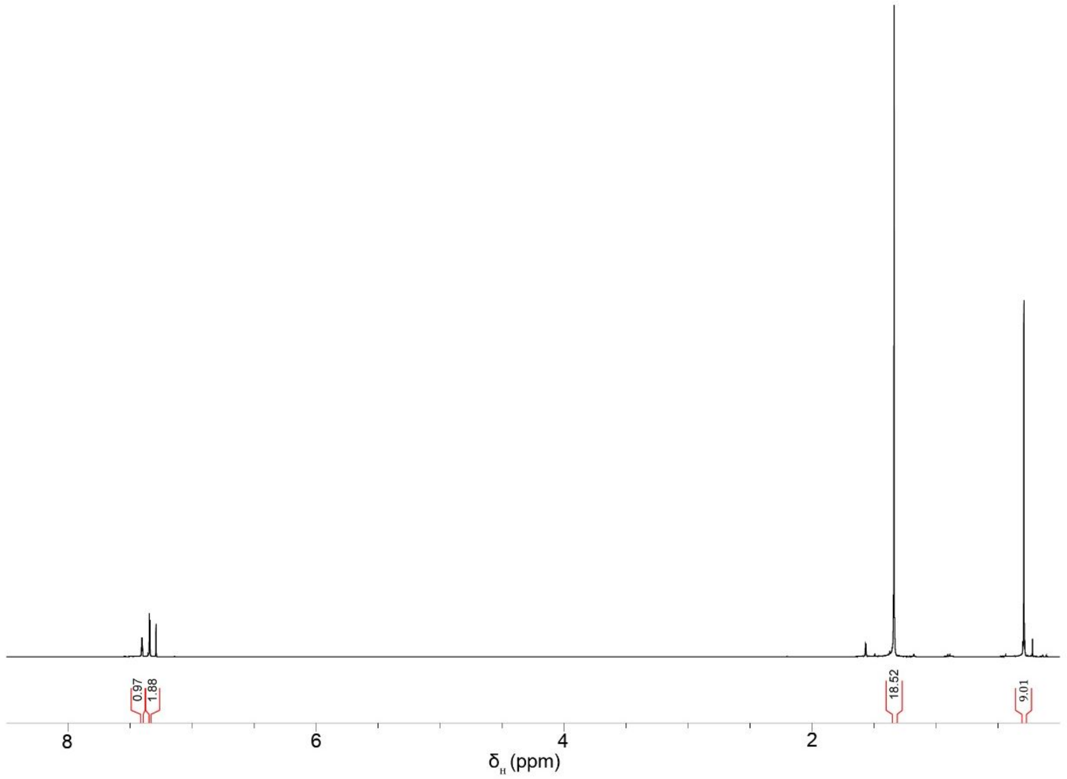

Figure S34. ^{1}H NMR (400 MHz, $CDCl_3$, 298 K).

S5 1,3-Di-*tert*-butyl-5-ethynylbenzene

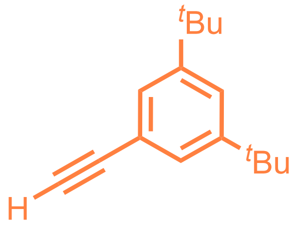

((3,5-Di-*tert*-butylphenyl)ethynyl)trimethylsilane (17.05 g, 59.5 mmol) and potassium carbonate (18.81 g, 136 mmol) in dichloromethane (25 mL) and methanol (25 mL) was stirred at room temperature for 16 hours. The reaction was diluted with dichloromethane (500 mL), dried over magnesium sulfate, then filtered through a silica plug (23 g, SiO_2), and the solvent removed *in vacuo* to yield the product as a white solid (10.62 g, 83 %) which was used without further purification; ^{1}H NMR (300 MHz, $CDCl_3$): δ_H = 7.45 (t, 1H, *J* = 2.0 Hz, Ar**H**), 7.38 (s, 2H, *J* = 2.0 Hz, 2 × Ar**H**), 3.06 (s, 1H, -C≡C**H**), 1.35 (s, 18H, 2 × -C(C**H₃**)**₃**). The data is in agreement with the literature.[3]

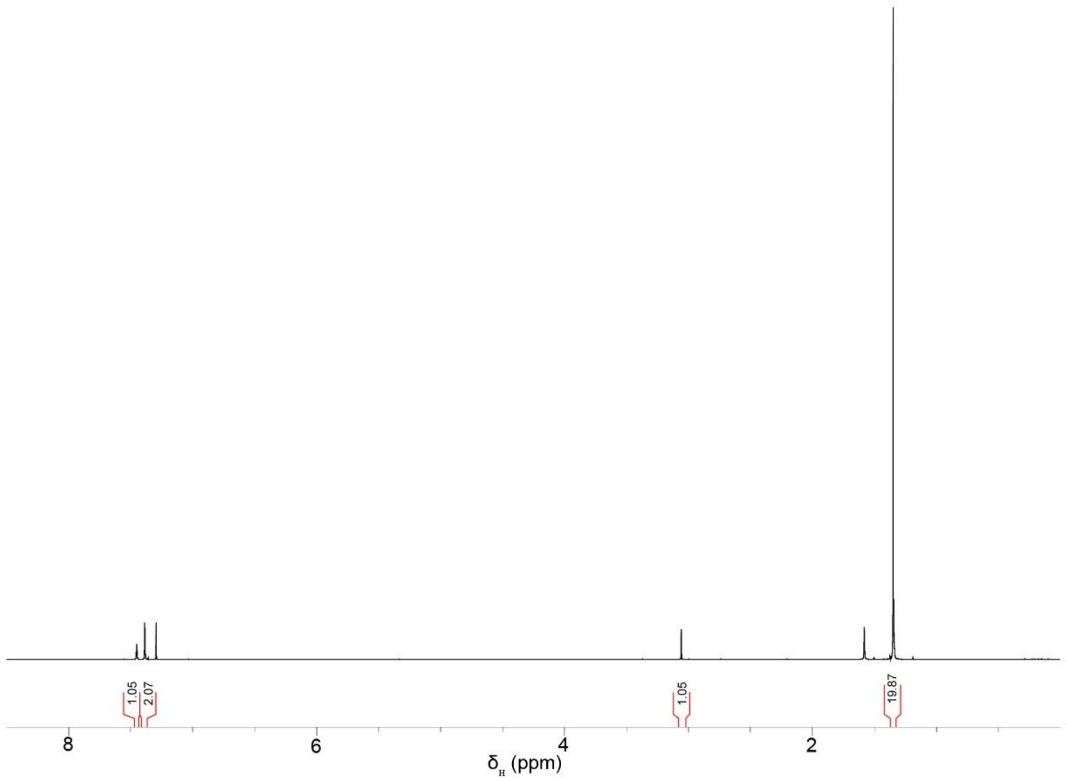

Figure S35. ^{1}H NMR (400 MHz, $CDCl_3$, 298 K).

S6 2-Amino-6-(dibutylamino)pyrimidin-4-ol

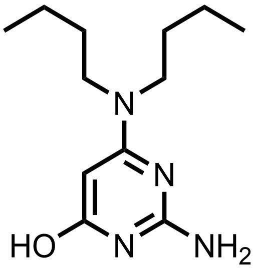

2- Amino-6-chloro-4-pyrimidinol hydrate (10.3 g, 70.8 mmol), dibutyl amine (23 mL, 177 mmol) in ethylene glycol (200 mL) was stirred at 135 ˚C for 16 hours. The reaction was allowed to cool, poured onto saturated sodium hydrogen carbonate (500 mL), extracted with ethyl acetate (2 × 500 mL), washed with saturated ammonium chloride (2 × 200 mL) 5 % w/v lithium chloride (2 × 200 mL), brine (200 mL), then dried over magnesium sulfate, filtered and the solvent removed *in vacuo* to yield the product as an off-white solid (14.0 g, 83 %); ^{1}H NMR (400 MHz, $CDCl_3$): δ_H = 12.46-12.20 (s, 1H, -O**H**) 5.30-5.10 (br s, 2H, -N$\mathbf{H_2}$), 4.78 (s, 1H, -C**H**), 3.30-3.20 (br t, 4H, *J* = 9.3 Hz, 2 ×-NC$\mathbf{H_2}$-), 1.51-1.42 (m, 4H, 2 ×-NCH_2C$\mathbf{H_2}$-), 1.29-1.19 (m, 4H, 2 × -C$\mathbf{H_2}$$CH_3$), 0.86 (t, 6H, *J* = 9.3 Hz, 2 × -C$\mathbf{H_3}$). The data is in agreement with the literature.[4]

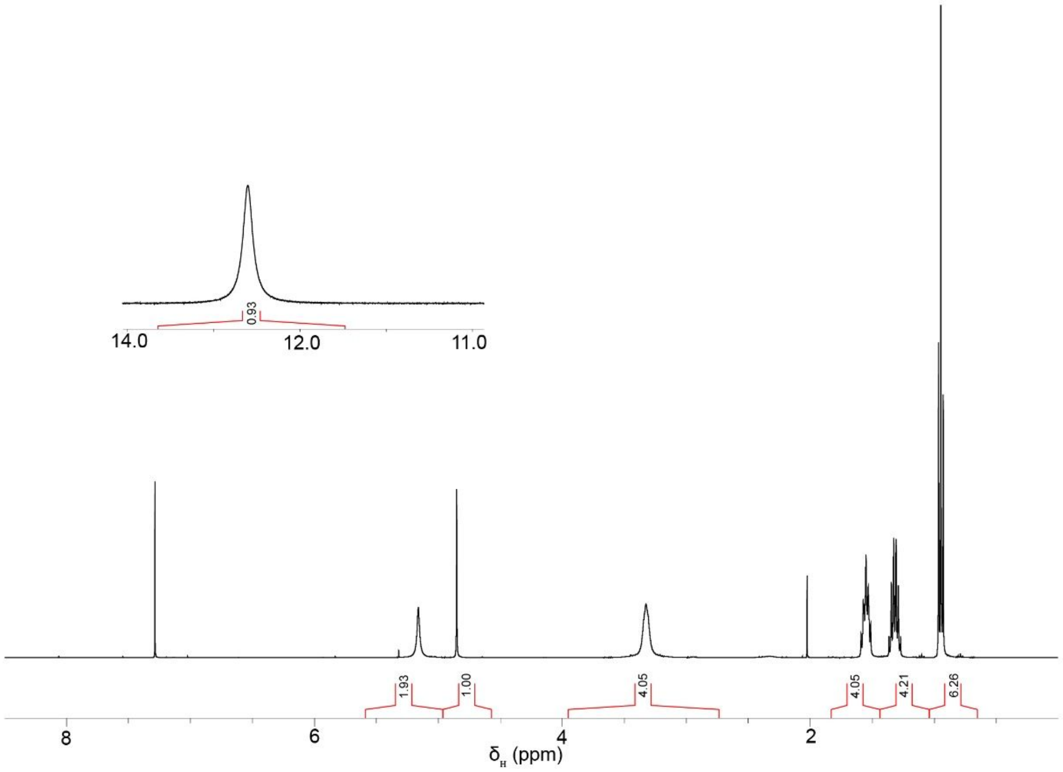

Figure S36. ^{1}H NMR (400 MHz, $CDCl_3$, 298 K).

4 *N*-[4-(dibutylamino)-6- (*tert*-butyl[diphenyl]silyl)oxypyrimidin-2-yl]-*N'*-butylurea

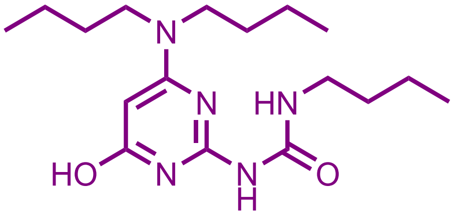

2-Amino-6-(dibutylamino)pyrimidin-4-ol (0.81 g, 3.39 mmol), *n*-butylisocyanate (0.58 mL, 5.15 mmol), *N,N*-dimethylamino pyridine (838 mg, 6.86 mmol), in dry chloroform (25 ml), was stirred at 70 ˚C, for 36 hours, protected by a nitrogen atmosphere. The reaction was allowed to cool, then the solvent removed *in vacuo*, and the resultant residue purified by crystallization from boiling acetone (30 mL), to yield the product as a white solid (0.29 g, 25 %); ^{1}H NMR (500 MHz, $CDCl_3$): δ_H = 12.64 (s, 1H, -O**H**), 11.25 (s, 1H, -N**H**), 9.62 (s, 1H, -N**H**), 5.36 (s, 1H, -C**H**), 3.43-3.28 (m, 6H, 3 × -NC**H₂**-), 1.66-1.55 (m, 6H, 3 × -NCH_2C**H₂**-), 1.45-1.33 (m, 6H, 3 × -C**H₂**CH_3), 1.01-0.94 (m, 9H, 3 × -C**H₃**). The data is in agreement with the literature.[4]

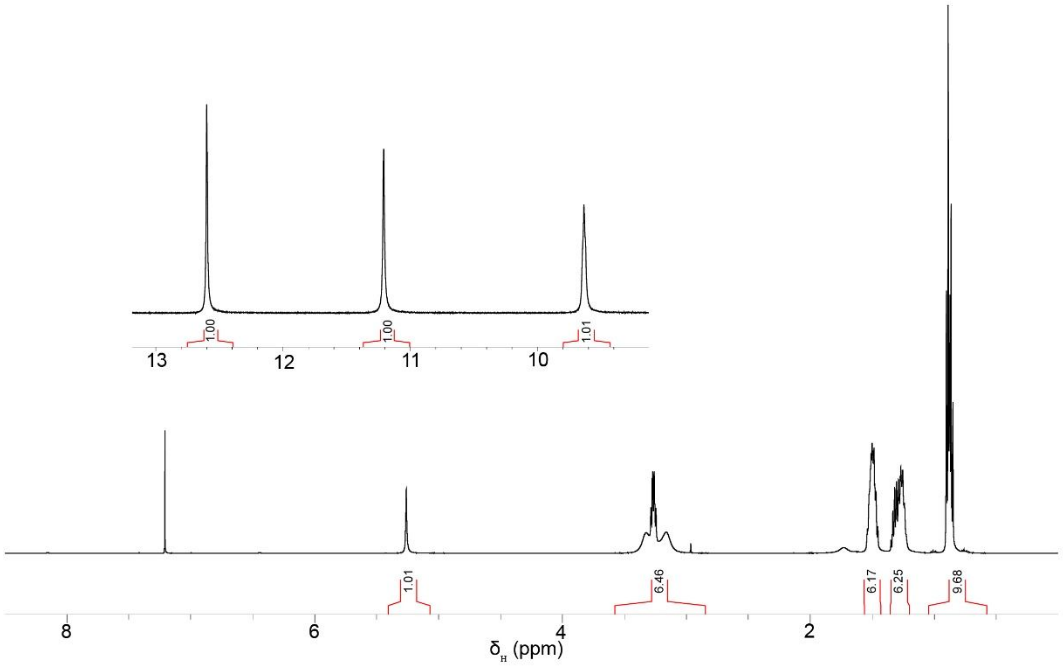

Figure S37. ^{1}H NMR (500 MHz, $CDCl_3$, 298 K).

S7 85:15 Ethyl 4-ethyl-3-oxooctanoate-Ethyl (2*Z*)-4-ethyl-3-hydroxyoct-2-enoate (assumed enol geometry)

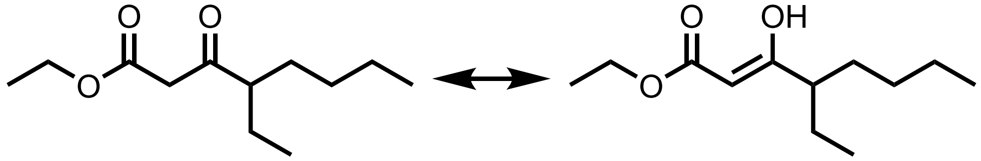

Potassium ethyl malonate (14.4 g, 84.5 mmol), magnesium chloride (9.61 g, 101 mmol) in dry acetonitrile (400 mL) was cooled to -10 ˚C. Triethylamine (18 mL, 129 mmol) was added in a single addition and the reaction was stirred vigorously, with warming to room temperature, for 2 hours, protected by a nitrogen atmosphere. The reaction was cooled to 0 ˚C, 2-ethylhexanoyl chloride (7.0 mL, 40.4 mmol) was added dropwise over 1 minute, and the reaction was stirred vigorously, with warming to room temperature, for 72 hours, under a nitrogen atmosphere. The solvent was removed *in vacuo*, and the residue was suspended in 6 M hydrochloric acid (200 mL), extracted with 40/60 petroleum ether (2 × 200 mL), dried over magnesium sulfate, filtered and the solvent removed *in vacuo* to yield the product as a clear oil (7.85 g, 91 %, 85:15 keto-enol ratio); ^{1}H NMR (300 MHz, $CDCl_3$): δ_H = 12.11 (0.15H, s, 1H, *enol* -O**H**), 4.98 (0.15H, s, 1H, *enol* –C=C**H**), 4.30-4.11 (s, 2H, -OC$\mathbf{H_2}$$CH_3$), 3.47 (1.70, s, 2H, *keto* (C=O)C$\mathbf{H_2}$(C=O)), 2.58-2.44 (m, 1H, -C**H**$(CH_2)_2$-), 1.67-1.24 (m, 11H, 4 × -C$\mathbf{H_2}$**-**, -OC$\mathbf{H_2}$$CH_3$), 0.95-0.86 (m, 6H, 2 × -C$\mathbf{H_3}$). The data is in agreement with the literature.[7]

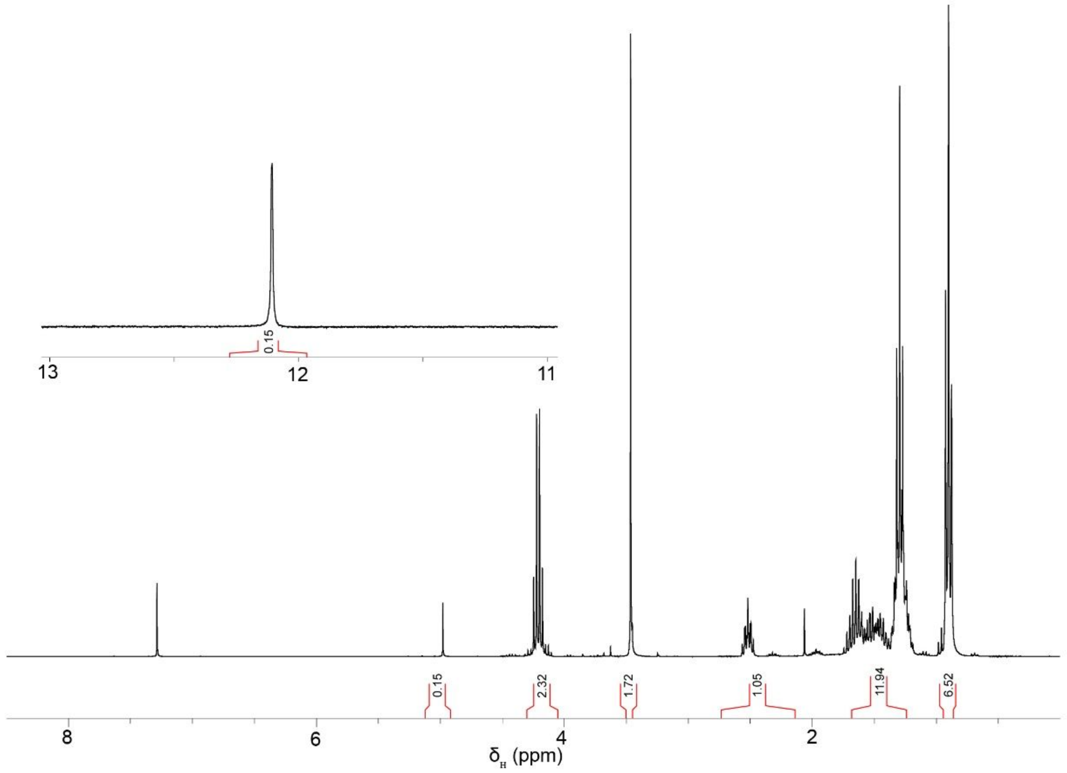

Figure S38. ^{1}H NMR (300 MHz, $CDCl_3$, 298 K).

S8 2-Amino-6-(heptan-3-yl)pyrimidin-4(1*H*)-one

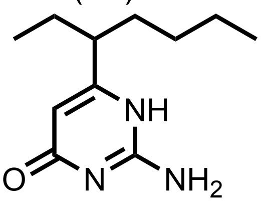

85:15 Ethyl 4-ethyl-3-oxooctanoate - ethyl (2*Z*)-4-ethyl-3-hydroxyoct-2-enoate (assumed enol geometry) (5.74 g, 24.1 mmol) and guanidinium carbonate (4.34 g, 24.1 mmol) in ethanol (100 mL) was stirred at reflux for 72 hours. The reaction was allowed to cool, the solvent removed *in vacuo*, and then the residue was suspended in saturated sodium hydrogen carbonate (100 mL), extracted with chloroform (2 × 200 mL), dried over magnesium sulfate, filtered and the solvent removed *in vacuo.* The resultant oily solid was stirred vigorously in *n*-pentane (200 mL) for 10 minutes and the insoluble pale yellow solid was filtered off to yield the product (3.24 g, 64 %[*1]);^{1}H NMR (300 MHz, $CDCl_3$): δ_H =7.26-6.22 (s, 2H, -N**H₂**), 5.61 (s, 1H, (O=C)C**H**-), 2.31-2.11 (m, 1H, -C**H**(CH_2)₂-), 1.69-1.44 (m, 4H, 2 × -C**H₂**-), 1.41-1.10 (m, 4H, 2 × -C**H₂**-), 0.91-0.78 (m, 6H, 2 × -C**H₃**). The data is in agreement with the literature.[6]

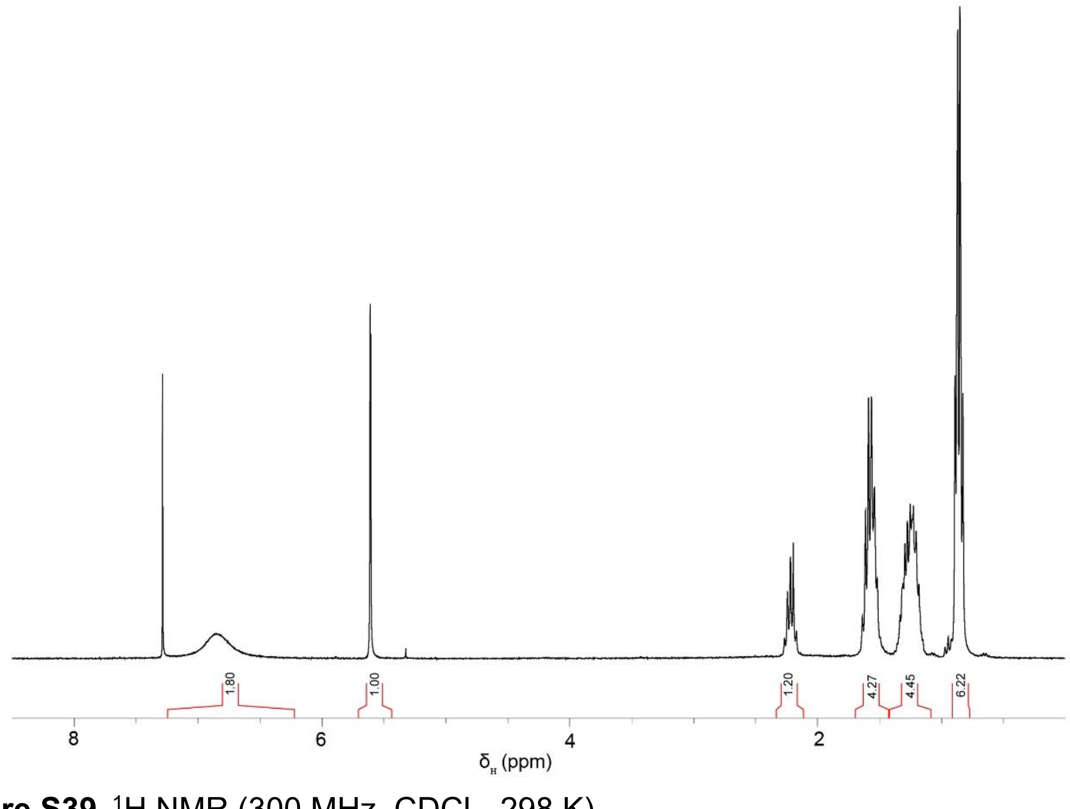

Figure S39. ^{1}H NMR (300 MHz, $CDCl_3$, 298 K).

[*1] The reaction has variable yields ranging from 16-64%. The *n*-pentane filtrate can have the solvent removed in *vacuo* to recover any unreacted keto-enol.

S9 7-Amino-1,8-Naphthyridin-2(1*H*)-one (Assumed tautomer)

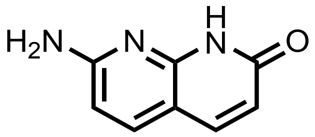

Concentrated sulfuric acid (40 mL) was added over 2 minutes to 2,6-diaminopyridine (6.66 g, 61.0 mmol) and 2-hydroxy-(2*S*)-butanedioic acid (9.00 g, 67.1 mmol) at 0 ˚C. The reaction was then stirred at 110 ˚C for 4 hours. The reaction was allowed to cool, and saturated ammonium hydroxide (~300 mL) added until the solution was pH = 9. The solid was filtered off and washed with water (200 mL) then diethyl ether (200 mL) to yield the product as an olive green solid (8.45 g, 86 %); ^{1}H NMR (500 MHz, d_6-DMSO): δ_H = 11.69-11.47 (br s, 1H, -N**H**), 7.63 (2 × d, 2H, *J* = 9.0 Hz, 2 × Ar**H**), 6.87-6.73 (br s, 2H, -N**H$_2$**), 6.32 (d, 1H, *J* = 9.0 Hz, Ar**H**), 6.09 (d, 1H, *J* = 9.0 Hz, Ar**H**). The data is in agreement with the literature. [4]

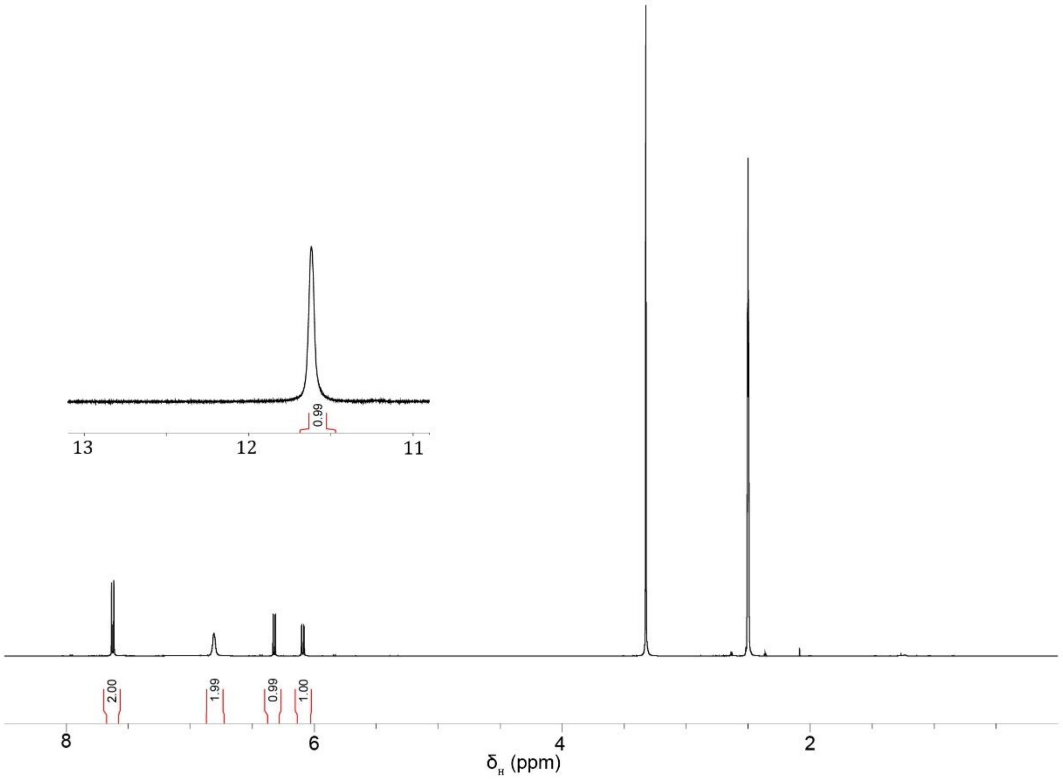

Figure S40. ^{1}H NMR (500 MHz, d_6-DMSO, 298 K).

S10 *N*-(7,8-Dihydro-7-oxo-1,8-naphthyridin-2-yl)-2-ethyl-hexanamide

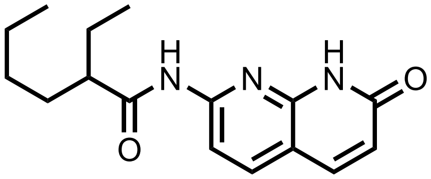

7-Amino-1,8-Naphthyridin-2(1*H*)-one (4.18 g, 26.0 mmol) and 2-ethylhexanoyl chloride (6.30 mL, 36.3 mmol) in pyridine (20 mL) was stirred at 110 ˚C for 21 hours. The reaction was poured onto 0.5 M hydrochloric acid (200 mL), extracted with dichloromethane (2 × 200 mL), and the combined organic layers were washed with saturated sodium hydrogen carbonate (200 mL), dried over magnesium sulfate, filtered, and the solvent removed *in vacuo*. The product was then purified by crystallization from boiling acetone (~200 mL) to yield the product as a brown solid (6.95 g, 93 %); ^{1}H NMR (500 MHz, $CDCl_3$): δ_H = 12.65 (s, 1H, -N**H**), 11.84 (s, 1H, -N**H**), 8.41 (d, 1H *J* = 8.6 Hz, Ar**H**), 7.91 (d, 1H *J* = 8.6 Hz, Ar**H**), 7.31 (d, 1H *J* = 9.4 Hz, Ar**H**), 6.60 (dd, 1H, *J* = 8.6 Hz, *J* = 1.4 Hz, Ar**H**), 7.68 (app. sept., 1H, *J* = 4.5 Hz, -C**H**(CH_2)$_2$-), 1.69-1.60 (m, 2H, -C**H₂-**), 1.59-1.41 (m, 2H, -C**H₂-**), 1.31-1.18 (m, 4H, 2 × -C**H₂**), 0.90 (t, 3H, *J* = 7.4 Hz, one of –C**H₃**), 0.79 (t, 3H, *J* = 7.2 Hz, one of –C**H₃**). The data is in agreement with the literature.[4]

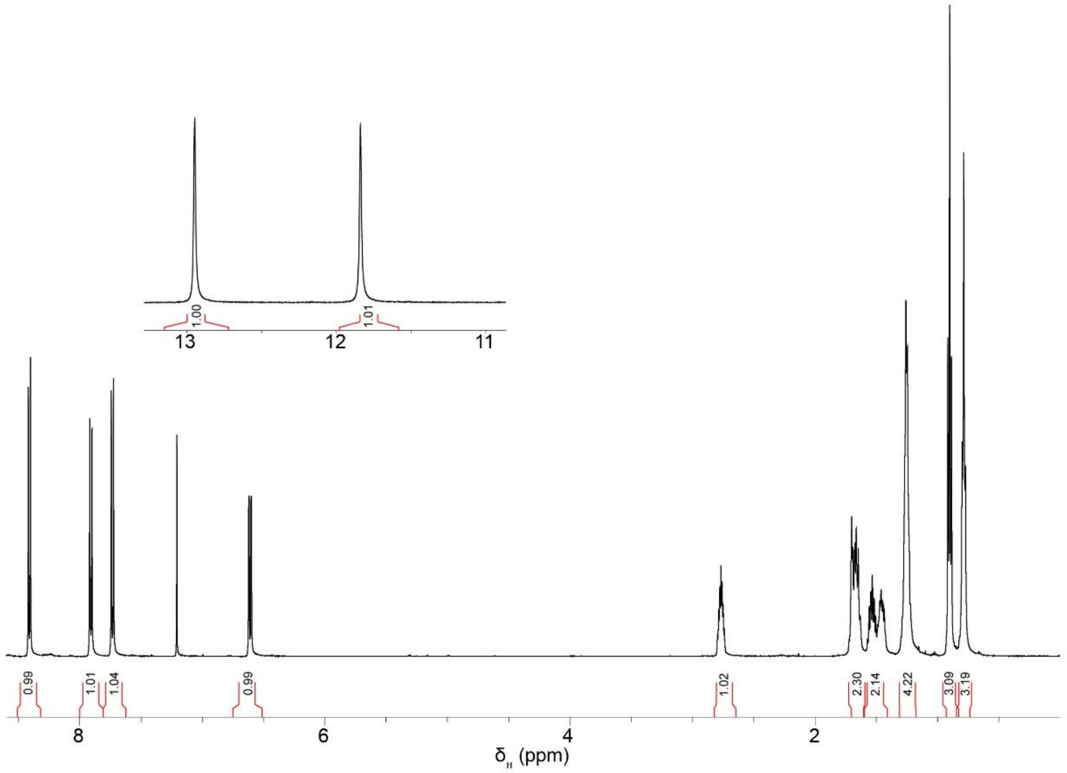

Figure S41. ^{1}H NMR (300 MHz, $CDCl_3$, 298 K).

S11 *N*-(7-chloro-1,8-naphthyridin-2-yl)-2-ethylhexanamide

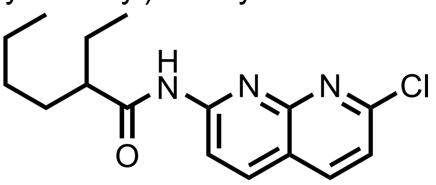

N-(7,8-Dihydro-7-oxo-1,8-naphthyridin-2-yl)-2-ethyl-hexanamide (2.94 g, 10.2 mmol) in phosphorous oxychloride (20 mL) was then stirred at reflux for 4 hours. The reaction was allowed to cool, carefully poured onto water (400 mL), took to pH 8-9 with saturated ammonia (~30 mL) and extracted with dichloromethane (2 × 200 mL). The combined organic extracts were washed with saturated sodium hydrogen carbonate (300 mL), brine (100 mL), dried over magnesium sulfate, filtered and the solvent removed *in vacuo* to yield the product as a brown glassy solid (2.48 g, 80 %); ^1H NMR (400 MHz, $CDCl_3$): δ_H = 8.92-8.74 (br s, 1H, -N**H**), 8.64 (d, 1H, *J* = 8.4 Hz, Ar**H**), 8.22 (d, 1H, *J* = 8.4 Hz, Ar**H**), 8.09 (d, 1H, *J* = 8.4 Hz, Ar**H**), 7.41 (d, 1H, *J* = 8.4 Hz, Ar**H**), 2.31 (app. sept., 1H, *J* = 4.5 Hz, -C**H**$(CH_2)_2$-), 1.82-1.50 (m, 4H, 2 × -C$\mathbf{H_2}$-), 1.39-1.29 (m, 4H, 2 × -C$\mathbf{H_2}$), 0.98 (t, 3H, *J* = 7.4 Hz, one of –C$\mathbf{H_3}$), 0.88 (t, 3H, *J* = 7.2 Hz, one of –C$\mathbf{H_3}$). The data is in agreement with the literature.[8]

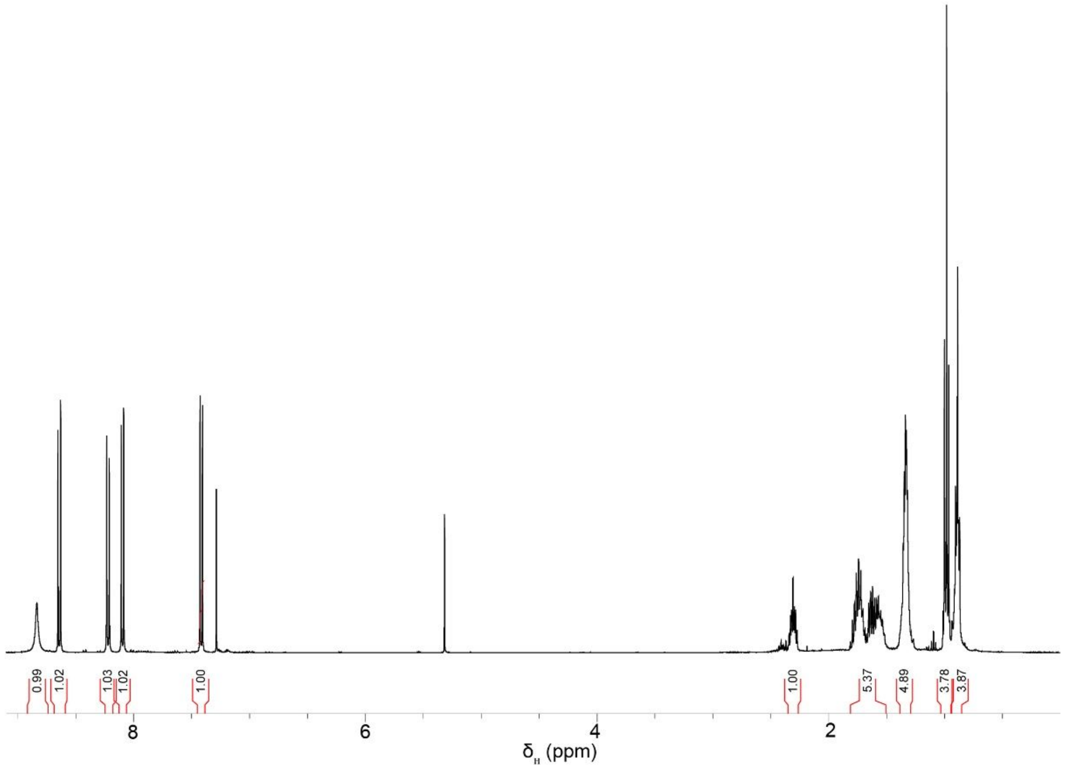

Figure S42. ^1H NMR (400 MHz, $CDCl_3$, 298 K).

6 *N,N'*-(1,8-naphthyridine-2,7-diyl)bis(2-ethylhexanamide) (1:1 mix of *meso* and chiral diastereoisomers)

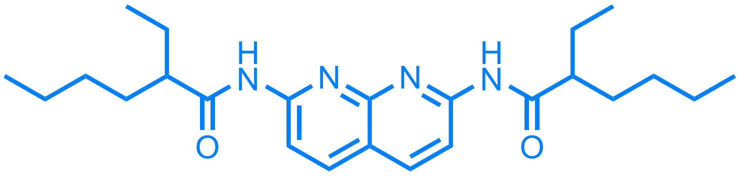

In an oven dried Schlenk flask *N*-(7-chloro-1,8-naphthyridin-2-yl)-2-ethylhexanamide (218 mg, 0.714 mmol), 2-ethylhexanamide (123 mg, 0.857 mmol), potassium carbonate (138 mg, 1.00 mmol), palladium acetate (8.0 mg, 0.0357 mmol), and 4,5-bis(diphenylphosphino)-9,9-dimethylxanthene (43.1 mg, 0.0714mmol), underwent 3 × evacuation-refill cycles. Under a strong flow of nitrogen dry 1,4-dioxane (5 mL) was added and the reaction mixture underwent 3 × freeze-evacuation-thaw-refill cycles. The reaction was stirred at 100 ˚C for 20 hours. The reaction was allowed to cool, diluted with ethyl acetate (200 mL), filtered through a Celite® plug, and had the solvent removed *in vacuo*. Following purification by manual flash chromatography (34 g silica, 80:10 chloroform-acetone) the product was isolated as a yellow solid (249 mg, 85 %); ^{1}H NMR (400 MHz, $CDCl_3$): δ_H = 8.42 (d, 2H, *J* = 8.9 Hz, 2 × Ar**H**), 8.22-8.02 (m, 2H, 2 × -N**H**), 8.08 (d, 2H, *J* = 8.9 Hz, 2 × Ar**H**), 2.22-2.10 (m, 2H, 2 ×-C**H**(CH_2)$_2$-), 1.74-1.46 (m, 8H, 4 × -C**H₂-**), 1.33-1.23 (m, 8H, 4 × -C**H₂-**), 0.92 (t, 6H, *J* = 7.2 Hz, 2 × –C**H₃**), 0.82 (t, 6H, *J* = 7.2 Hz, 2 × -C**H₃**). The data is in agreement with the literature.[5]

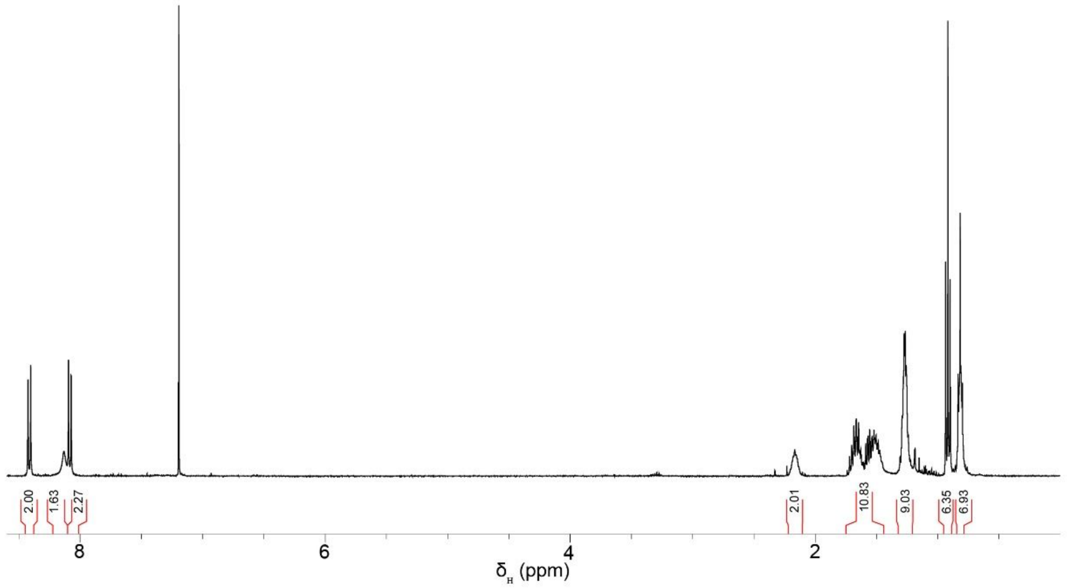

Figure S43. ^{1}H NMR (400 MHz, $CDCl_3$, 298 K).

References

(1) Lewis, J. E. M.; Bordoli, R. J.; Denis, M.; Fletcher, C. J.; Galli, M.; Neal, E. A.; Rochette, E. M.; Goldup, S. M. High Yielding Synthesis of 2,2′-Bipyridine Macrocycles, Versatile Intermediates in the Synthesis of Rotaxanes. *Chem. Sci.* **2016**, *7* (5), 3154–3161. https://doi.org/10.1039/C6SC00011H.

(2) Gupta, V.; Mandal, S. K. A Highly Stable Triazole-Functionalized Metal–Organic Framework Integrated with Exposed Metal Sites for Selective CO 2 Capture and Conversion. *Chem. – A Eur. J.* **2020**, *26* (12), 2658–2665. https://doi.org/10.1002/chem.201903912.

(3) Ogi, S.; Ikeda, T.; Wakabayashi, R.; Shinkai, S.; Takeuchi, M. A Bevel-Gear-Shaped Rotor Bearing a Double-Decker Porphyrin Complex. *Chem. - A Eur. J.* **2010**, *16* (28), 8285–8290. https://doi.org/10.1002/chem.201000276.

(4) Coubrough, H. M.; van der Lubbe, S. C. C.; Hetherington, K.; Minard, A.; Pask, C.; Howard, M. J.; Fonseca Guerra, C.; Wilson, A. J. Supramolecular Self-Sorting Networks Using Hydrogen-Bonding Motifs. *Chem. – A Eur. J.* **2019**, *25* (3), 785–795. https://doi.org/10.1002/chem.201804791.

(5) Ligthart, G. B. W. L.; Ohkawa, H.; Sijbesma, R. P.; Meijer, E. W. Pd-Catalyzed Amidation of 2-Chloro- and 2,7-Dichloro-1,8-Naphthyridines. *J. Org. Chem.* **2006**, *71* (1), 375–378. https://doi.org/10.1021/jo051864b.

(6) Keizer, H. M.; González, J. J.; Segura, M.; Prados, P.; Sijbesma, R. P.; Meijer, E. W.; de Mendoza, J. Self-Assembled Pentamers and Hexamers Linked through Quadruple-Hydrogen-Bonded 2-Ureido-4[1H]-Pyrimidinones. *Chem. - A Eur. J.* **2005**, *11* (16), 4602–4608. https://doi.org/10.1002/chem.200500329.

(7) Ma, Y.-Z.; Xiao, H.; Yang, X.-F.; Niu, L.-Y.; Wu, L.-Z.; Tung, C.-H.; Chen, Y.-Z.; Yang, Q.-Z. Bidirectional Singlet and Triplet Energy Transfer via the 2-Ureido-4[1 H]-Pyrimidinone Quadruple Hydrogen-Bonded Module. *J. Phys. Chem. C* **2016**, *120* (30), 16507–16515. https://doi.org/10.1021/acs.jpcc.6b05437.

(8) Ligthart, G. B. W. L.; Ohkawa, H.; Sijbesma, R. P.; Meijer, E. W. Complementary Quadruple Hydrogen Bonding in Supramolecular Copolymers. *J. Am. Chem. Soc.* **2005**, *127* (3), 810–811. https://doi.org/10.1021/ja043555t.
